# Cellular **α**-synuclein pathology is associated with bioenergetic dysfunction in Parkinson’s iPSC-derived dopamine neurons

**DOI:** 10.1093/hmg/ddz038

**Published:** 2019-02-11

**Authors:** Federico Zambon, Marta Cherubini, Hugo J R Fernandes, Charmaine Lang, Brent J Ryan, Viola Volpato, Nora Bengoa-Vergniory, Siv Vingill, Moustafa Attar, Heather D E Booth, Walther Haenseler, Jane Vowles, Rory Bowden, Caleb Webber, Sally A Cowley, Richard Wade-Martins

**Affiliations:** 1 The Oxford Parkinson’s Disease Centre, University of Oxford, Oxford, UK; 2 Department of Physiology, Anatomy and Genetics, University of Oxford, South Parks Road, Oxford, UK; 3 Wellcome Trust Centre for Human Genetics, University of Oxford, Oxford, UK; 4 Sir William Dunn School of Pathology, South Parks Road, Oxford, UK

## Abstract

Parkinson’s disease (PD) is the second most common neurodegenerative disorder and a central role for α-synuclein (αSyn; *SNCA*) in disease aetiology has been proposed based on genetics and neuropathology. To better understand the pathological mechanisms of αSyn, we generated induced pluripotent stem cells (iPSCs) from healthy individuals and PD patients carrying the A53T *SNCA* mutation or a triplication of the *SNCA* locus and differentiated them into dopaminergic neurons (DAns). iPSC-derived DAn from PD patients carrying either mutation showed increased intracellular αSyn accumulation, and DAns from patients carrying the *SNCA* triplication displayed oligomeric αSyn pathology and elevated αSyn extracellular release. Transcriptomic analysis of purified DAns revealed perturbations in expression of genes linked to mitochondrial function, consistent with observed reduction in mitochondrial respiration, impairment in mitochondrial membrane potential, aberrant mitochondrial morphology and decreased levels of phosphorylated DRP1^Ser616^. Parkinson’s iPSC-derived DAns showed increased endoplasmic reticulum stress and impairments in cholesterol and lipid homeostasis. Together, these data show a correlation between αSyn cellular pathology and deficits in metabolic and cellular bioenergetics in the pathology of PD.

## Introduction

Parkinson’s disease (PD) is the second most common neurodegenerative disorder with adulthood onset and is characterized by the preferential loss of dopaminergic neurons (DAns) in the *substantia nigra* pars compacta (*SN*pc) in the midbrain. The principal pathological hallmark of PD pathology is the presence of cytoplasmic inclusions in the surviving neurons, termed Lewy Bodies (LBs), composed predominantly of the protein α-synuclein (αSyn; *SNCA*) (1). Patients carrying mutations and copy number variants in *SNCA* represent very rare autosomal dominant forms of PD (2,3). The function of αSyn is not well understood and the underlying molecular mechanisms associated with the onset of PD are still unclear. αSyn oligomers that form during early stages of αSyn aggregation are thought to be highly toxic to many intracellular processes and organelles (4). Recent studies exploring the pathology associated with αSyn in PD have proposed a variety of mechanisms, including mitochondrial dysfunction (5), impairment of chaperone-mediated autophagy (6), lysosomal dysfunction (7), induction of endoplasmic reticulum (ER) stress (8,9) and functional interaction with fatty acid binding proteins (FABPs) (10).

Human induced pluripotent stem cells (iPSCs) preserving an individual’s genetic background can be generated from PD patients and combined with protocols for the differentiation of iPSCs into DAns to generate highly relevant cell models to study PD pathology *in vitro* (11). In this study, we carried out a thorough phenotypic analysis of DAns differentiated from nine independent iPSC lines, generated from three healthy individuals and four PD patients carrying either the A53T αSyn mutation (A53T *SNCA*) or a triplication of the *SNCA* locus (*SNCA* Tripl). We identified accumulation of αSyn in both A53T *SNCA* and *SNCA* Trip DAns, and an increase in the burden of αSyn oligomer load detected by the proximity ligation assay (αSyn-PLA) (12) and increased αSyn release in the extracellular medium in the *SNCA* Tripl DAn. RNA-sequencing (RNA-seq) analysis of purified DAns identified perturbations in pathways associated with mitochondrial dysfunction that was confirmed by decreases in basal respiration, maximal respiration and spare capacity, an impairment in mitochondrial membrane potential, aberrant mitochondrial morphology and a decrease in the levels of phosphorylated DRP1^Ser616^. Finally, induction of ER stress and perturbations in cellular lipid biology were detected in both A53T *SNCA* and *SNCA* Tripl DAns. Together, these data identify perturbations in protein accumulation, cellular metabolism and bioenergetics in the pathological mechanisms of PD and confirm the importance of patient stem cell models to study these pathways.

## Results

### Differentiation of control, A53T *SNCA* and *SNCA* triplication iPSCs to DAns

Independent iPSC lines were generated from three healthy individuals (control) and three PD patients carrying the A53T αSyn mutation (A53T *SNCA*). Three iPSC clonal lines were also derived from a single PD patient with an *SNCA* triplication (*SNCA* Tripl) (Supplementary Material, Fig. S1A). Multiple iPSC lines were studied to account for biological variability, and quality control analyses of all the iPSC lines have been recently described (13). Here, we differentiated iPSC lines to iPSC-derived DAns as we described previously (14). Feeder-free iPSC cultures remained positive for the pluripotency marker Oct3/4 (15), and 11 days after neuronal induction most cells were positive for the ventral midbrain markers FOXA2 and LMX1A, confirming efficient midbrain patterning (16). By 22 days in vitro (DIV), most cells were confirmed neurons by expression of β3-tubulin (TUJ1) with a high proportion of tyrosine hydroxylase (TH) positive neurons (Supplementary Material, Fig. S1B). By 35 DIV, an extensive neuronal network of TUJ1^+^ and TH^+^ cells was visible and some TH^+^ cells co-expressed the ventral midbrain markers FOXA2 and LMX1A (16) (Supplementary Material, Fig. S2A). Differentiation efficiency was assessed by TUJ1, TH and FOXA2 expression using immunocytochemistry. No difference in differentiation efficiency was detected across genotypes with an average of 77% neurons (TUJ1^+^) and 44% DAns (TH^+^) cells, of which 70% co-expressed FOXA2^+^ (Supplementary Material, Fig. S2B and C).

### **α**Syn accumulation, oligomerization and secretion in PD *SNCA* iPSC-derived DAns

**Figure 1:**
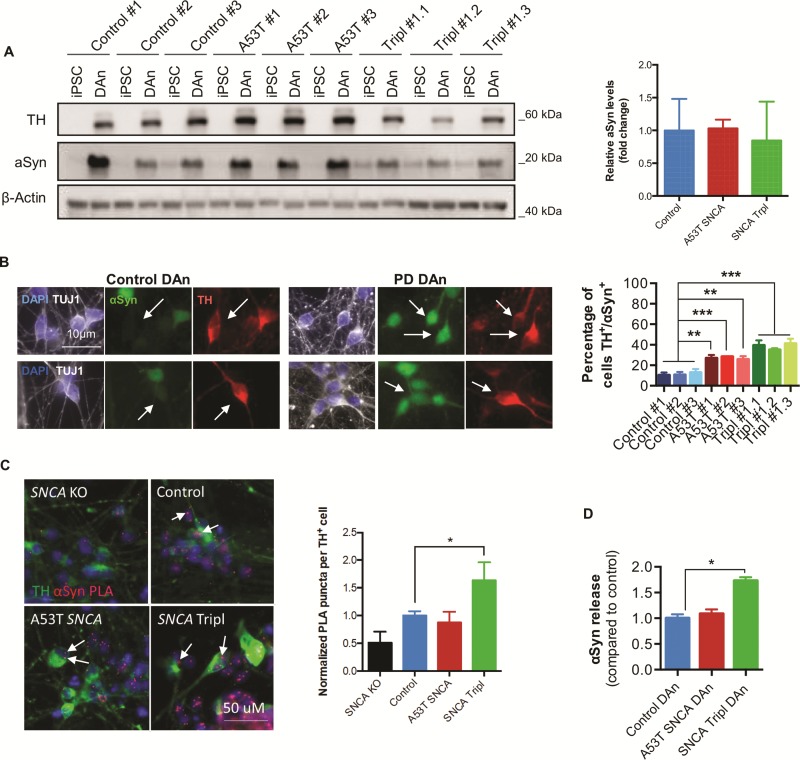
αSyn accumulation, oligomerization and secretion in PD iPSC-derived DAns. (**A**) Quantification of αSyn protein levels by Western blot (N = 3, mean ± SEM). (**B**) Representative images of TUJ1, TH and αSyn immunocytochemistry and quantification of the percentage of TH^+^ cells with intracellular αSyn (N = 3, mean ± SEM, one-way ANOVA; ^**^P < 0.01, ^***^P < 0.001, ^****^P < 0.0001). (**C**) Representative image of αSyn-PLA and TH immunocytochemistry and quantification of the average number of PLA puncta in TH^+^ cells (white arrows). Each bar represents the mean ± SD number of PLA puncta in at least 25 individual TH^+^ cells per line (one-way ANOVA, ^*^P < 0.05). (**D**) Quantification of αSyn concentration in culture medium by MSD platform. Each bar represents the mean ± SEM of differentiated lines from three independent individuals or clonal lines per genotype (one-way ANOVA, ^*^P < 0.05).

αSyn is a natively unfolded protein expressed in neurons (17) and is thought to be involved in pathological mechanisms leading to neurodegeneration of midbrain DAns in PD. Quantification of total intracellular monomeric αSyn protein levels and analysis of the percentage of total αSyn^+^ cells revealed no differences between genotypes (Fig. 1A; Supplementary Material, Fig. S3A and B). However, immunocytochemical analysis revealed that a higher proportion of TH^+^ cells accumulated intracellular αSyn in A53T *SNCA* and *SNCA* Tripl DAns compared to control neurons (Fig. 1B). To follow up on our previous observations on the presence of oligomer pathology in the brain and probe its importance in a patient cellular model, we assessed the oligomeric state of the protein using the αSyn-PLA (12) integrated with immunocytochemistry for TH to restrict the analysis to DAns. Specificity of the αSyn-PLA signal was confirmed by differentiating an isogenic iPSC *SNCA* knockout (KO) line that lacks the expression of αSyn into DAns (Supplementary Material, Fig. S3C) and also by removing components of the proximity ligation assay, which resulted in no observed signal (Supplementary Material, Fig. S3D). An increase in the average number of αSyn-PLA puncta was detected in the cell body of TH^+^ cells in the *SNCA* Tripl DAns compared to controls, suggesting increased αSyn oligomerization (Fig. 1C). Previously, we have shown an increase in the secretion of αSyn in iPSC-derived DAns from PD patients carrying the N370S *GBA* mutation (18). Analysis of αSyn release in the culture medium from *SNCA* iPSC-derived DAn cultures at the later stages of the neuronal maturation revealed increased αSyn release in *SNCA* Tripl, but not A53T *SNCA* DAns, compared to control (Fig. 1D). This increase in αSyn release was not due to increased cellular toxicity and independent of non-specific protein release by cells (Supplementary Material, Fig. S4).

### Transcriptomic analysis of purified TH^+^ cells reveals perturbations in mitochondria function in A53T *SNCA* and *SNCA* Tripl iPSC-derived DAns

To investigate the pathological mechanisms associated with the presence of the A53T *SNCA* mutation or the *SNCA* Tripl copy number variants in DAns, TH^+^ cells from iPSC-derived DAn cultures were purified by fluorescence-activated cell sorting (FACS) as described previously (14). No statistically significant differences were detected across genotypes in terms of the total number of TH+ cells collected, the percentage of TH^+^ cells within the population and the quality of the extracted RNA (RNA integrity, RIN > 9) (Supplementary Material, Fig. S5).

**Figure 2:**
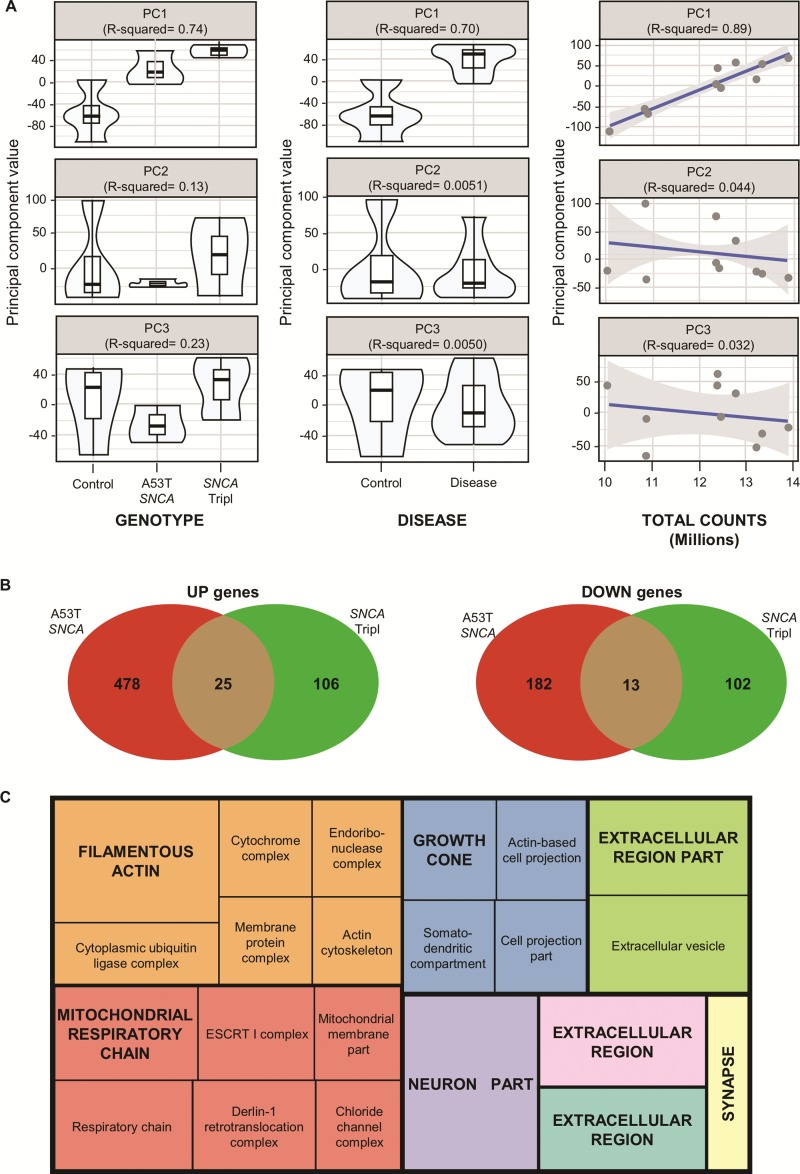
RNA-seq analysis of purified iPSC-derived A53T *SNCA* and *SNCA* Tripl DAns. (**A**) The first three principal components scores (from PCA plots in Supplementary Material, Fig. S6) are plotted against known covariates and the R^2^ value from a linear model regressing each PC onto the variable of interest is reported. (**B**) Common and distinct DE genes between the comparisons of the A53T *SNCA* and *SNCA* Tripl lines to control (upregulated genes on the left, downregulated genes on the right). (**C**) Most representative enriched GOCC terms among 38 overlapping DE genes between the comparisons of the A53T *SNCA* and *SNCA* Tripl lines to control (derived using ReviGO) (54). Colours represent main non-redundant GO term sets (see Materials and Methods) and size is related to the statistical significance of enrichment (absolute log10 *P*-value).

Following RNA-seq, principal component analysis (PCA) on the set of expressed 14 214 coding genes (counts, >1) demonstrated that samples clustered by genotype and disease along the first principal component achieving R^2^ correlations of 0.74 and 0.70, respectively, which indicates a good separation of the groups correlating to disease (Fig. 2A; Supplementary Material, Fig. S6A). We also found that the total number of sample gene counts was also well correlated with the first principal component (R^2^ = 0.89). To detect differentially expressed (DE) genes, we applied DESeq2 with a model correcting for known covariates, i.e. total sample counts, gender, age and sorting day that correlate with variation captured by PCA (Supplementary Material, Fig. S6). In total, 698 DE genes were detected in the A53T *SNCA* iPSC-derived DAns and 246 DE genes in the *SNCA* Tripl iPSC-derived DAns, as compared to control iPSC-derived DAns. Of the 52 genes differentially expressed in both *SNCA* genotypes as compared to control, 38 genes showed consistent directionality (up/downregulated) and 14 DE genes were in a different direction in each mutation compared to control (Fig. 2B). The DE genes in the A53T *SNCA* iPSC-derived DAns were associated with mitochondria-related Gene Ontology (GO) terms, while the DE genes in the *SNCA* Tripl iPSC-derived DAns were mainly characterized by synaptic transmission and histone acetylation pathways (Supplementary Material, Fig. S7).

Overall, the 38 genes differentially expressed in both genotypes in a consistent direction showed a significant enrichment in mitochondria-, ER- and endosome-related GO Cellular Component terms (Fig. 2C; Supplementary Material, Table S1 for complete list of enriched GO terms).

### Impaired mitochondrial function in A53T *SNCA* and SNCA Tripl iPSC-derived DAns

**Figure 3:**
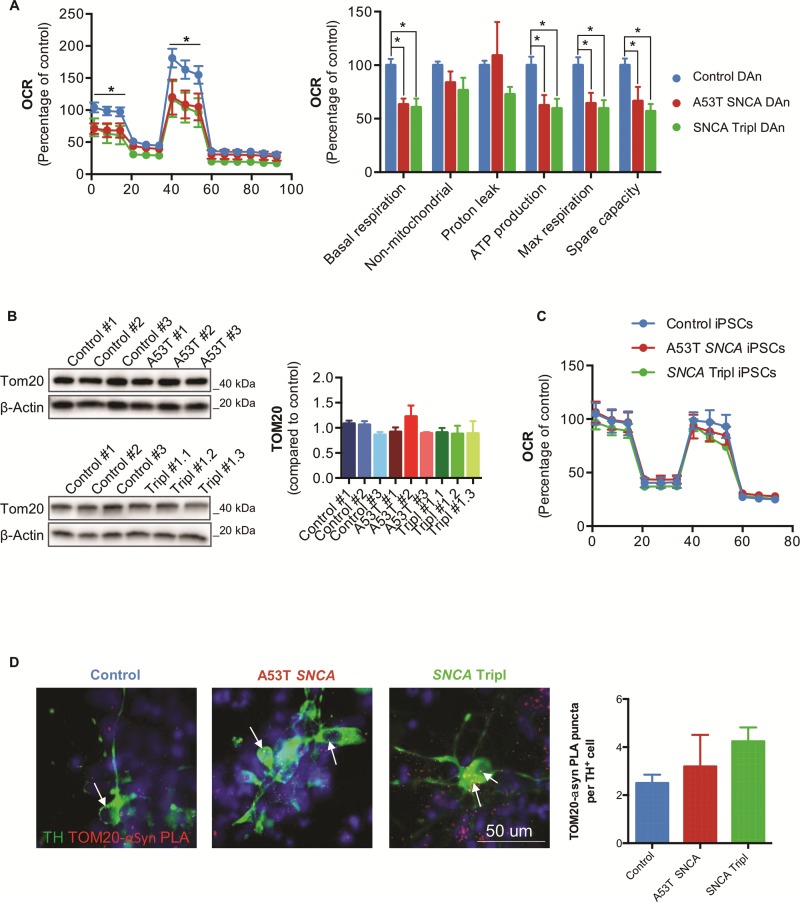
Mitochondrial dysfunction in iPSC-derived A53T *SNCA* and *SNCA* Tripl DAns. (**A**) OCRs and mitochondria respiration parameters normalized to cellular protein content in DAns (N = 3, mean ± SEM, Student’s *t*-test, ^*^P < 0.05). (**B**) Quantification of TOM20 expression by Western blot in DAns (N = 3, mean ± SEM). (**C**) OCR traces of undifferentiated iPSCs (N = 3, mean ± SEM). (D) Representative images and quantification of TOM20-αSyn-PLA in TH^+^ neurons. Each bar represents mean ± SEM number of PLA puncta (white arrows) in at least 25 individual TH^+^ neurons per line.

We wished to explore the bioenergetic consequences of the accumulation of αSyn species and the observed differential expression of mitochondria-related genes in PD *SNCA* iPSC-derived DAns. We first used the Seahorse XFe96 Flux Analyzer platform to examine the cellular bioenergetics of PD DAns compared to controls. PD DAns were found to have a decrease in basal respiration (35% for A53T *SNCA* and 40% for *SNCA* Tripl), maximal respiration (35% for A53T *SNCA* and 30% for *SNCA* Tripl), spare capacity (30% for A53T *SNCA* and 30% for *SNCA* Tripl) and ATP production (35% for A53T *SNCA* and 30% for *SNCA* Tripl) compared to control DAns (Fig. 3A), suggesting impaired mitochondrial function. This difference was not due to changes in total mitochondrial mass as shown by the quantification of the mitochondrial marker TOM20 (Fig. 3B). No significant differences were found in glycolytic activity as measured by the extracellular acidification rate (ECAR) (Supplementary Material, Fig. S8A). Undifferentiated iPSCs did not show differences in oxygen consumption rate (OCR) across genotypes (Fig. 3C). We also confirmed that neuronal cells rely more on mitochondrial respiration than glycolysis, as described by the higher OCR/ECAR ratio and spare capacity (Supplementary Material, Fig. S8B and C). Finally, we probed whether αSyn and TOM20 interact in iPSC-derived DAns, thereby testing whether there is a direct link between αSyn and the mitochondrial membrane. The αSyn-TOM20 PLA showed puncta in control, A53T *SNCA* and *SNCA* Tripl patient iPSC-derived DAns, indicating an interaction between these proteins in DAns (Fig. 3D). Removing components of the proximity ligation assay as a control resulted in no signal (Supplementary Material, Fig. S8D).

### Mitochondria dysfunction in A53T *SNCA* and *SNCA* Tripl iPSC-derived DAns is associated with changes in mitochondria morphology and membrane potential

**Figure 4:**
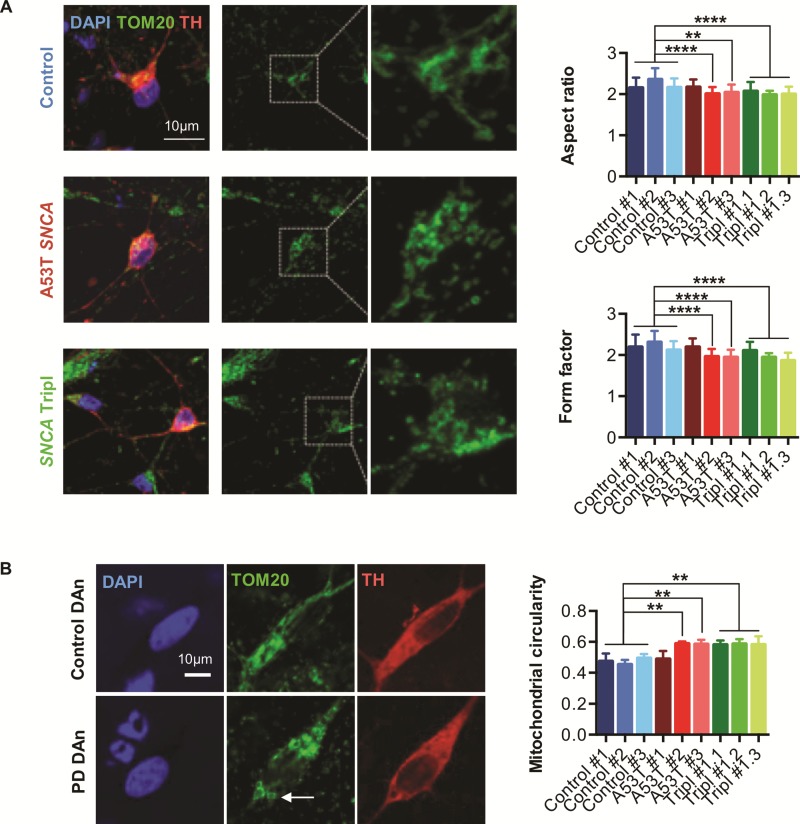
Abnormal mitochondrial morphology and decreased mitochondrial membrane potential in iPSC-derived A53T *SNCA* and *SNCA* Tripl DAns. (**A**) Representative images and quantification of aspect ratio and form factor to describe mitochondrial morphology in DAns. (**B**) Representative and quantification of mitochondrial circularity to describe donut-shaped mitochondria (white arrow). In each case, data represent the mean ± SEM from three independent differentiation (N = 3). One-way ANOVA, ^**^P < 0.01, ^***^P < 0.001, ^****^P < 0.0001.

Mitochondria are highly dynamic organelles, constantly dividing and fusing to exert their function at specific cellular locations (19). Importantly, abnormal changes in mitochondrial shape have been linked to αSyn oligomers and aggregates (20,21) indicating a mechanistic link between αSyn and mitochondrial morphology in PD. To better understand the mitochondrial dysfunction in A53T *SNCA* and *SNCA* Tripl iPSC-derived DAns, we assessed mitochondrial morphology using confocal microscopy by co-staining for TH and the mitochondrial marker TOM20 (22). The shape descriptors `aspect ratio (AR)’ and `form factor (FF)’ describe mitochondrial circularity and complexity, respectively, and lower values are associated with circular and unbranched mitochondria. Both AR and FF were significantly decreased in TH+ cells, indicating more rounded and less branched mitochondria in two of three A53T *SNCA* and in all *SNCA* Tripl DAns (Fig. 4A). In addition, we observed circular donut-shaped mitochondria, previously seen in response to oxidative stress (23), only in A53T *SNCA* and *SNCA* Tripl iPSC-derived DAns (Fig. 4B).

**Figure 5:**
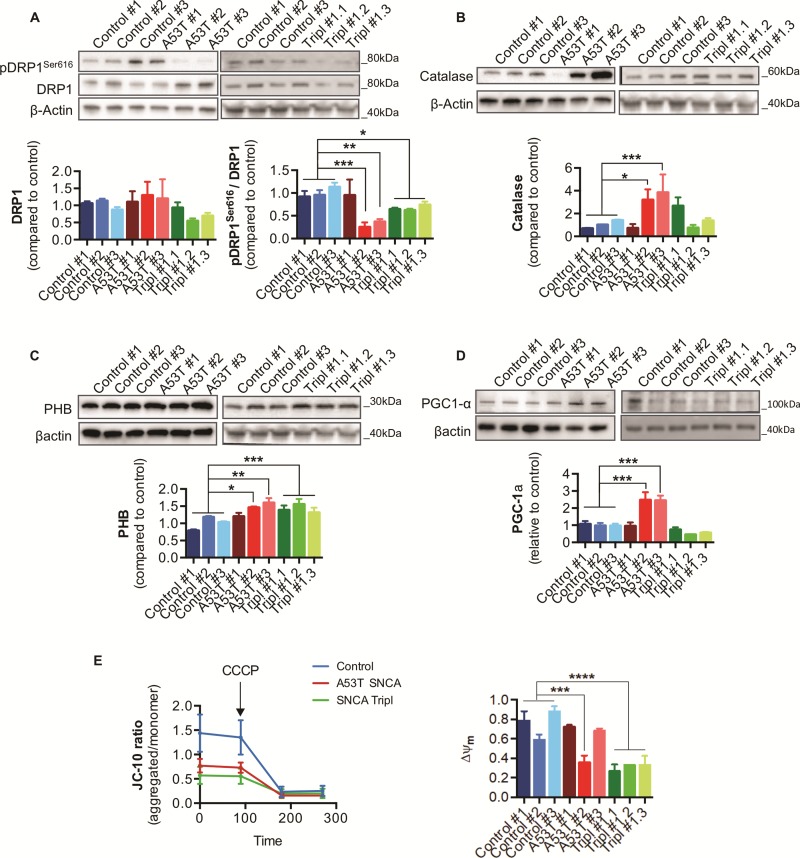
Changes in proteins involved in mitochondrial homeostasis and response to ROS production in iPSC-derived A53T *SNCA* and *SNCA* Tripl DAns. (**A**) DRP1 and phosphorylated DRP1^Ser616^ protein levels in DAns. (**B**) Catalase protein levels in DAns. (**C**) PHB protein levels in DAns. (**D**) PGC1-α protein levels in DAns. (**E**) Quantification of mitochondrial membrane potential using JC-10 in DAns. In each case, data represent the mean ± SEM from three independent differentiations (N = 3). One-way ANOVA, ^*^P < 0.05, ^**^P < 0.01.

Since mitochondrial oxidative stress causes imbalance in mitochondrial fission–fusion (24), we measured the protein levels of regulators of mitochondrial dynamics. Levels of the phosphorylated mitochondrial fission dynamin-1-like protein (pDRP1^Ser616^) were reduced in two A53T *SNCA* and in all *SNCA* Tripl iPSC-derived DAns (Fig. 5A), indicating a shift towards mitochondrial fission in PD DAns. Moreover, both the long and short isoform of the mitochondrial protein dynamin-like 120 kDa protein (L-OPA1 and S-OPA1, respectively) and mitofusin 2 (MFN2), proteins known to regulate mitochondrial fusion, were not significantly different between genotypes (Supplementary Material, Fig. S9A and B). We found that levels of catalase, a major cellular antioxidant enzyme responsible for detoxifying reactive oxygen species (ROS), were found significantly increased in iPSC-derived DAns from two A53T *SNCA* lines compared to control (Fig. 5B). Expression levels of prohibitin (PHB), a mitochondrial protein associated with mitochondrial functional integrity and control of ROS production by the NADH dehydrogenase (25,26), were increased in PD DAns (Fig. 5C), whereas the regulator of mitochondrial biogenesis peroxisome proliferator-activated receptor gamma coactivator 1-α (PGC1-α) (27), also known to activate anti-oxidant enzymes, was found to be increased only in A53T *SNCA* DAns (Fig. 5D), potentially as a compensatory mechanism in response to increased mitochondrial damage and oxidative stress (28).

Finally, to confirm the respiratory defects in *SNCA* iPSC-derived DAns, we assessed the mitochondrial membrane potential (Ψ_m_), which is also crucial for the regulation of mitochondrial dynamics. Using a ratiometric dye, JC-10, we found that A53T *SNCA* #2 and all *SNCA* Tripl DAns exhibit reduced Ψ_m_ compared to control cells (Fig. 5E), confirming the compromised mitochondrial function.

### ER stress and protein homeostasis perturbations in A53T *SNCA* and *SNCA* Tripl iPSC-derived DAns

**Figure 6:**
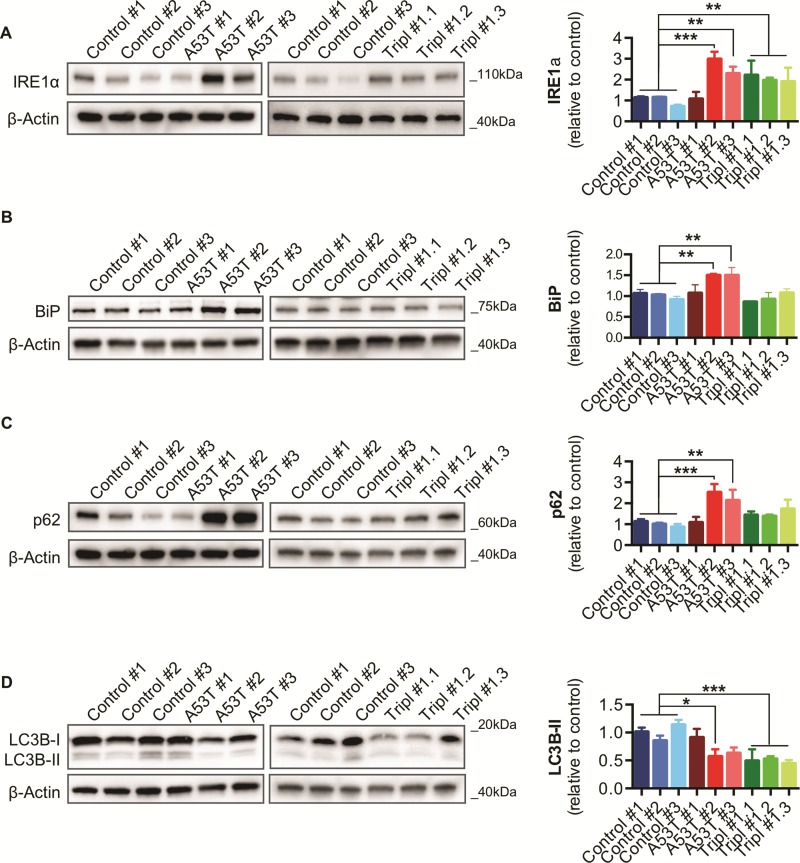
Upregulation of ER stress markers and autophagic dysfunction in iPSC-derived A53T *SNCA* and *SNCA* Tripl DAns. (**A**) BiP protein levels in DAns. (**B**) IRE1α protein levels in DAns. (**C**) p62 protein levels in DAns. (**D**) LC3-I and LC3-II protein levels in DAns. In each case, data represent the mean ± SEM from three independent differentiation (N = 3). One-way ANOVA, ^*^P < 0.05, ^**^P < 0.01.

Previous studies have reported increased ER stress in heterozygous N370S *GBA* DAns (18) and A53T *SNCA* iPSC-derived cortical neurons (8), which may be linked to αSyn oligomerization (9,29). An increase in protein levels of IRE1α, a mediator of the unfolded protein response, confirmed ER stress activation in DAns from two A53T *SNCA* and all *SNCA* Tripl lines (Fig. 6A). Analysis of ER chaperones revealed a significant increase in the expression of BiP/GRP78 (Fig. 6B), but not protein disulfide isomerase (PDI) or calreticulin (Supplementary Material, Fig. S10A, B), in DAns from two A53T *SNCA* lines compared to controls. ER stress has also been linked to perturbations in autophagy (18,30) and DAns from two A53T *SNCA* lines showed a significant increase in p62 levels (Fig. 6C). DAns from A53T *SNCA* #2 and all *SNCA* Tripl lines also had decreased processing of the autophagosome marker LC3 to its lipidated form LC3-II (Fig. 6D). No significant differences were seen in the levels of the lysosomal marker LAMP1 and the chaperone-mediated autophagy (CMA) markers LAMP2A and HSC70 (Supplementary Material, Fig. S10C–E), despite A53T αSyn being previously shown to affect the CMA markers LAMP2A and HSC70 (6). This may indicate that lysosomal dysfunction is not a prominent phenotype of A53T *SNCA* and *SNCA* Trip DAns, at least at this stage of the neuronal maturation.

### Dysregulation of lipid homeostasis and bioenergetics in A53T *SNCA* and *SNCA* Tripl iPSC-derived DAns

**Figure 7:**
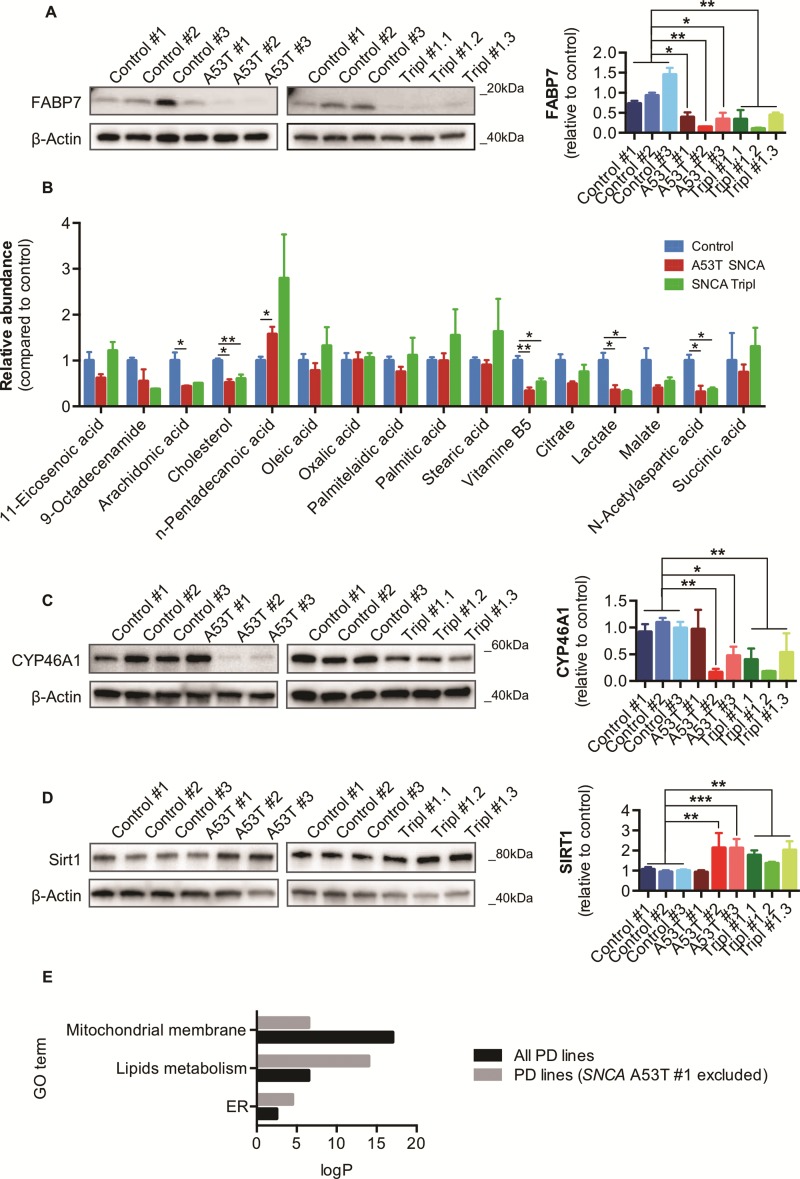
Dysregulation of lipid homeostasis and metabolism in iPSC-derived A53T *SNCA* and *SNCA* Tripl DAns. (**A**) FABP7 protein levels in DAns (N = 3, mean ± SEM, one-way ANOVA, ^*^P < 0.05, ^**^P < 0.01). (**B**) Metabolomics analysis of DAns (N = 1, three independent lines or clones per genotype, mean ± SEM, one-way ANOVA, ^*^P < 0.05, ^**^P < 0.01). (**C**) CYP46A1 protein levels in DAns (N = 3, mean ± SEM, one-way ANOVA, ^*^P < 0.05, ^**^P < 0.01). (**D**) SIRT1 protein levels in DAns (N = 3, mean ± SEM, one-way ANOVA, ^**^P < 0.01, ^***^P < 0.001). (**E**) Pathway analysis for the two sets of DE genes between control and PD lines (with or without A53T *SNCA* #1) focused on phenotypes characterized in the previous sections (ER-, lipids metabolism- and mitochondria-related terms). The significance of enrichment is reported on y axis (absolute log10 *P*-value) and colours represent different sets of DE genes.

The primary sequence of αSyn contains a motif shared with fatty acid binding proteins (FABPs) (31), which regulates the intracellular flux of lipids and are central mediators of metabolism and cell signalling (32). We found FABP7, recently reported as having reduced expression in the striatum and *substantia nigra* in PD brain (33), to be strongly decreased in PD iPSC-derived DAns compared to control (Fig. 7A). To further explore the possibility of impaired fatty acid metabolism in PD DAns we performed a metabolomic analysis (34) across genotypes and identified multiple metabolites involved in mitochondria respiration and lipid metabolism. Several metabolites involved in mitochondrial and fatty acid metabolism were altered in PD DAns, including lactate, N-acetylaspartic acid and pantothenic acid in both A53T *SNCA* and *SNCA* Tripl DAns (Fig. 7B). In particular, the metabolomics analysis highlighted a significant decrease in the level of cholesterol in PD DAns. In agreement, we found reduced levels of the protein cholesterol 24-hydroxylase (CYP46A1), an ER enzyme involved in the regulation of cholesterol metabolism in neurons (35), in DAns from two A53T *SNCA* and all *SNCA* Tripl lines (Fig. 7C). Lastly, the level of Sirtuin1 (SIRT1), a deacetylase which regulates the activation of transcription factors involved in cellular metabolism, including PGC1-α, and metabolic pathways including the metabolism of glucose and lipids (36), was upregulated in PD DAns compared to controls (Fig. 7D).

In several analyses, DAns from the A53T *SNCA* #1 line appeared phenotypically distinct from the other A53T *SNCA* lines and not representative of its genotype. To explore the impact of this on the study, we removed this line and grouped the two remaining A53T *SNCA* and all *SNCA* Tripl samples together to compare to controls for differential gene expression analysis. Following the removal of A53T *SNCA* #1, the number of genes found to be differentially expressed increased from 1503 (all 6 PD lines versus control) to 2534 (5 PD lines versus control). This larger set included 98% of the 1503 DE genes and 47% of the 38 genes that were consistently differentially expressed in DAns from the A53T *SNCA* and *SNCA* Tripl lines when compared to controls (Supplementary Material, Fig. S11A). Together, these results suggested that the transcriptomic variation DAns from the A53T *SNCA* #1 line was discordant with the variation in the remaining lines. As many new DE genes were identified following the removal of A53T *SNCA* #1, we also analysed differences at the pathway level focusing on GO terms related to phenotypes described in the previous sections (Fig. 7E). Indeed, this new analysis identified an enrichment in lipid metabolism and ER terms that were less over-represented in the previous analysis which included A53T *SNCA* #1 (Fig. 7E; Supplementary Material, Fig. S11B).

## Discussion

In this work, we used iPSC-derived DAns from PD patients carrying the A53T *SNCA* or the *SNCA* Tripl mutation to study the cellular mechanisms associated with αSyn in PD pathology. We found that TH^+^ cells in PD *SNCA* iPSC-derived DAns displayed intracellular accumulation of αSyn and that *SNCA* Tripl mutation DAns exhibited an increased burden of oligomeric αSyn species as shown by the αSyn-PLA (12) and elevated αSyn release. αSyn accumulation and oligomerization have been shown to be a hallmark of PD (1,12) and play a causative role in mitochondrial dysfunction in *in vitro* PD models (37). We therefore further investigated the consequences of αSyn pathology in our human *in vitro* model of PD.

We sought to investigate the effect of the *SNCA* mutations and αSyn pathology at the transcriptional level, specifically in TH^+^ cells. Transcriptomics analysis of purified TH^+^ iPSC-derived DAns found a dysregulation in many mitochondrial genes in the PD neurons. Analysis of mitochondrial activity showed that αSyn accumulation and oligomerization was associated with a reduction in basal respiration, maximal respiration and spare capacity in PD DAn cultures. We also observed changes in mitochondrial morphology in TH^+^ cells of A53T *SNCA* and *SNCA* Tripl iPSC-derived DAns, which were associated with a decreased phosphorylation of DRP1^Ser616^. Interestingly, the decrease in phosphorylation of DRP1^Ser616^ may shift mitochondrial dynamics towards fission (38), which, in turn, could explain the respiration deficits seen in PD DAns. Particularly, we observed donut-shaped mitochondria, which are a hallmark of high oxidative stress (23,39) in the *SNCA* iPSC-derived DAn. In line with this, levels of catalase and PHB, both prominent intracellular ROS scavengers, were also increased, alongside PGC1α which activates antioxidant enzymes, all of which taken together potentially link the observed mitochondria dysfunction with ROS production (26). It is also interesting that we were able to confirm in iPSC-derived DAns a previously described interaction between αSyn and TOM20 in post-mortem PD brain (40), which has been proposed to be a cause of mitochondrial defects in PD. It is possible that an increased or aberrant association between αSyn and mitochondria could constitute a possible mechanism underlying mitochondrial dysfunction in the PD *SNCA* DAns.

We observed that that A53T *SNCA* and *SNCA* Tripl DAns have increased levels of IRE1α, one of the effectors of the ER stress pathway, whereas the ER chaperone BiP was upregulated only in A53T *SNCA* neurons. ER stress is an emerging pathological mechanism associated with PD (29) and has been associated with the presence of mutated αSyn and oligomers (9,41) and mitochondrial dysfunction (41). Our data show the association of αSyn pathology with the activation of at least one of the ER stress pathways and the marked reduction in mitochondrial function in PD DAns. We also found an increase of p62 in A53T *SNCA* DaNs and a decrease in the levels of LC3B-II in at least one of the A53T *SNCA* lines and in *SNCA* Tripl DAns, which is comparable with other reports describing the effects of αSyn accumulation on macroautophagy (42).

A recent detailed and systematic analysis of multiple PD brain regions highlighted mitochondrial perturbations and lipid transport defects as the major protein alterations found in PD patient brains (33). Interestingly, FABP7 was recently proposed as a potential biomarker for early PD (33) and our results for reduced expression of FABP7 in PD DAns are in agreement with this hypothesis. We also assessed the metabolic perturbations linked to mitochondrial dysfunction and found a marked reduction in cholesterol levels in our PD iPSC-derived DAns. We further confirmed a reduction in the expression levels of CYP46A1, an ER enzyme crucial for cholesterol metabolism. The brain is the most cholesterol-rich organ in the human body and defects in cholesterol metabolism have been associated with neurodegenerative diseases including PD (43). SIRT1 is a NAD^+^-dependent deacetylase involved in a wide variety of metabolic functions including mitochondria respiration and lipid metabolism (44). SIRT1 levels were found increased in A53T *SNCA* and *SNCA* Tripl DAns, together with an upregulation of one of its targets PGC1-α in A53T *SNCA* DAns, suggesting that SIRT1 could be an important regulator of the observed bioenergetic perturbations in these lines.

Overall, a regulatory overlap between the described ER stress, mitochondria respiration and lipid metabolism pathways in A53T *SNCA* and *SNCA* Tripl DAns is likely and the mechanisms and implications of such pathological interactions need to be further explored. Due to the putative localization of αSyn at the mitochondria-associated membranes (MAMs), the pathological mechanism may occur at these intracellular microdomains (45). Dysfunction in lipid homeostasis and induction of ER stress may also be a result of αSyn oligomerization at the MAMs or ER, which are the main sites of lipid synthesis (46,47).

Because of increased αSyn accumulation, oligomerization and release in our PD DAn cultures, we propose an association between the genetic mutations and αSyn pathology with the cellular dysfunction reported in this work. All iPSC lines were generated using highly standardized conditions and characterized for their similar differentiation efficiency. Therefore, it is likely that these phenotypes are a consequence of pathological mechanisms rather than, for example, a different nature or maturation stage of the TH^+^ cells among lines or genotypes. Moreover, we were able to confirm phenotypes in multiple iPSC lines with different *SNCA* genotypes. Interestingly, DAns from A53T *SNCA* #1 displayed only a few phenotypes compared to the other PD lines, including increased cytoplasmic αSyn staining, mitochondrial dysfunction and decreased FABP7 expression. Also, when this line was removed from the transcriptomic analysis, a more robust phenotype in terms of ER stress and disruption of lipids metabolism was reported, indicating that this line may not be representative of its genotype. The A53T *SNCA* mutation is estimated to have a penetrance of ~85% (48), which may explain variability at the cellular level in the case of A53T *SNCA* #1 DAns. Nonetheless, phenotypic variability among patient-derived iPSC lines with the same genotype has been documented before (18,49).

In summary, our results combining cell biology and transcriptomics suggest that αSyn cellular pathology (increased accumulation, oligomer formation and release) in *SNCA* PD DAns are associated with mitochondrial dysfunction, ER stress and impaired cholesterol and lipid metabolism. The analysis of both DAns and undifferentiated iPSCs from nine different iPSC lines and clones from four PD patients and three healthy individuals supports the hypothesis that aSyn cellular pathology impacts cellular bioenergetics in dopamine neurons, which may be the cause of cytotoxicity leading to cell loss of DAns in PD. Our work highlights the importance of using iPSC-derived DAns from PD patients to study αSyn pathology in a physiologically relevant *in vitro* cell model of PD.

## Materials and Methods

### Culture of iPSC and differentiation to DAns

The generation and characterization of iPSCs from healthy individuals or PD patients carrying the A53T *SNCA* mutation or a triplication of the *SNCA* locus were described in a previous study (13). Feeder-free iPSC lines were routinely cultured on Matrigel (BD Biosciences) in mTeSR1 (StemCell Technologies). Differentiation was carried as described previously (16), with slight modifications (14,50) (see Supplemental Experimental Procedures). All experiments were carried out at 35 DIV (i.e. 15 days of maturation after the replating of the cells at 20 DIV) and at least 3 independent differentiations.

### Immunocytochemistry and Western blot

These procedures were performed using standard methods and details are given in Supplemental Experimental Procedures.

### Measurement of mitochondria respiration

Mitochondrial respiration and glycolytic activity of DAns were measured using a Seahorse XFe96 Analyzer (Seahorse Bioscience) and normalized to total well protein content (see Supplemental Experimental Procedures).

### PLA for **α**Syn oligomers and image acquisition

Cells were immuno-stained for TH and subsequently αSyn oligomeric species were detected as described previously but using the αSyn 4D6 antibody (Abcam ab1903) (12). A total of 60X z-stacks were captured on an EVOS FL Auto Cell Imaging System (Life Technologies) and analysed using ImageJ (NIH, Bethesda, Maryland, USA). The TH channels were used to generate masks to analyse the PLA signal in individual TH^+^ cells.

### Detection of **α**Syn in supernatant

The medium was spun at 1000*g* for 5 min to remove cell debris. αSyn content was determined using the Meso Scale Diagnostic (MSD) Human α-Synuclein Kit (K151TGD-2) following manufacturer’s instructions. The absolute αSyn concentration was calculated with the DISCOVERY WORKBENCH® analysis software based on the standard curve. Data were normalized to the protein content of the corresponding well that the supernatant was collected from. Adenylate kinase activity was also measured in identical samples using ToxiLight cytotoxicity assay (Lonza; LT07–217).

### Measurement of mitochondrial membrane potential

To detect changes in mitochondrial membrane potential (ΔΨm), cells were washed with Krebs buffer (145 mm NaCl, 5 mm KCl, 10 mm HEPES, 1 mm MgCl_2_, 1 mm CaCl_2_, 5.6 mm glucose and pH 7.4/NaOH) and loaded with 5 μm JC-10 (a derivative of 5,5′,6,6′-tetrachloro-1,1,3,3′-tetraethyl-benzimidazolyl-carbocyanine iodide, JC-1; AAT Bioquest, Inc.) at 37°C for 30 min. The fluorescence intensities were measured using the multi-mode plate reader PHERAstar FSX (BMG Labtech) by fluorescence excitation/emission maxima: 514/529 nm, monomer form, and 585/590 nm, aggregate form. To establish that the JC-10 signal was indicative of ΔΨm, experiments were terminated inducing a maximal mitochondrial depolarization by addition of 10 μm carbonyl cyanide 3-chlorophenylhydrazone (CCCP; Sigma-Aldrich).

### Assessment of mitochondrial morphology by confocal microscopy

Z-stack images were collected at 0.4 μm intervals by an Olympus FV1000 laser scanning confocal microscope using a 100× numerical aperture objective with a 3× digital zoom and standard pinhole. Quantitative analysis of mitochondrial morphology was performed using ImageJ software as previously described (22). Briefly, raw images were de-convolved, thresholded and mitochondrial morphological features were quantified. These were the aspect ratio, which reflects the `length-to-width ratio’ (AR, the ratio between the major and minor axis of the ellipse equivalent to the mitochondrion); the form factor, which describes the complexity and branching aspect of mitochondria (FF, defined as Pm^2^/4πAm, where Pm is the length of mitochondrial outline and Am is the area of mitochondrion); and circularity, (defined as 4π·Am/Pm^2^), a dimensional index of sphericity.

### FACS of TH^+^ cells from DAn cultures and RNA extraction for RNA-seq

TH^+^ cells from DAn cultures were purified using FACS prior RNA extraction for RNA sequencing as previously described (14) (see Supplemental Experimental Procedures).

### RNA-seq and bioinformatics analysis

Sequencing was carried on an Illumina Hiseq4000 obtaining 75PE reads. Basic quality control screenings on unmapped reads and sequence mapping were performed through CGAT pipeline pipeline_readqc.py. The quality of the sequencing was assessed by FASTQC software (version 0.9.3), (http://www.bioinformatics.babraham.ac.uk/projects/fastqc/). RNA-seq data were mapped to the hg19 assembly via STAR version 2.2.0c (51). Read alignments were merged in single BAM file output per sample. Reads were filtered to remove those not uniquely mapped (mapping quality equal to 255) and all ribosomal and mitochondrial RNA reads. Gene-level read counts were obtained using FeatureCount (52). The list of 20 157 protein coding genes was obtained from the UCSC genome browser for the hg19 assembly. No outliers are identified based on number and expression of coding genes (Supplementary Material, Fig. S12). All analyses are performed on a set of 14.214 coding genes expressed in at least half of the samples. DE genes were estimated using DESeq2 R package (53) for disease effect correcting for the experimental structure (i.e. design formula defined as `~ sorting_day + gender + age + genotype + disease + genotype:disease ’) at FDR of ≤0.1. A classical enrichment analysis was performed by testing the over-representation of gene ontology biological processes (GO BP) and cellular components (GO CC) terms within the group of DE genes using a Fisher test (TopGO R package from Alexa, A. and Rahnenfuhrer, J. (2016), topGO: Enrichment Analysis for Gene Ontology). REVIGO (54) was used to obtain the most representative enriched GO terms based on semantic similarity (*simRel* score). PCA is performed on log transformed gene counts through prcomp R function (center = T,scale = T).

### Statistical analysis

All statistical analysis and graphical representation was performed in Prism version 6 (GraphPad software). Specific tests are noted in each figure legend. Experimental results were analysed usingo one-way analysis of variance (ANOVA) with Dunnet’s multiple comparison test and *P-*values of less than 0.05 were considered statistically significant. Data from at least three multiple independent differentiations were analysed. Each individual A53T *SNCA* line and the average of the three clones of the *SNCA* Tripl line were compared to the average of control DAns.

## Supplementary Material

Supplementary Data
