# Supplementary material for: Cellular α-synuclein pathology is associated with bioenergetic dysfunction in Parkinson’s iPSC-derived dopamine neurons
Source: Hum Mol Genet. 2019 Feb 11;28(12):2001–13. doi: 10.1093/hmg/ddz038 (PMC6548224; doi:10.1093/hmg/ddz038)
Supplement: Supplementary Data [file suppl_data_ddz038.zip › Suppl Figures and Table.pdf]

A

iPSC line	Clone	In this study	Genotype	Age	Gender
SFC841-03	1	Control #1	Control	36	Male
SFC840-03	6	Control #2		67	Female
SFC856-03	4	Control #3		78	Female
SFC828-03	6	A53T *SNCA* #1	A53T *SNCA*	51	Female
SFC829-03	6	A53T *SNCA* #2		46	Male
SFC830-04	9	A53T *SNCA* #3		51	Male
SFC831-03	1	*SNCA* Tripl #1	*SNCA* Tripl	55	Female
	3	*SNCA* Tripl #2			
	5	*SNCA* Tripl #3			

B

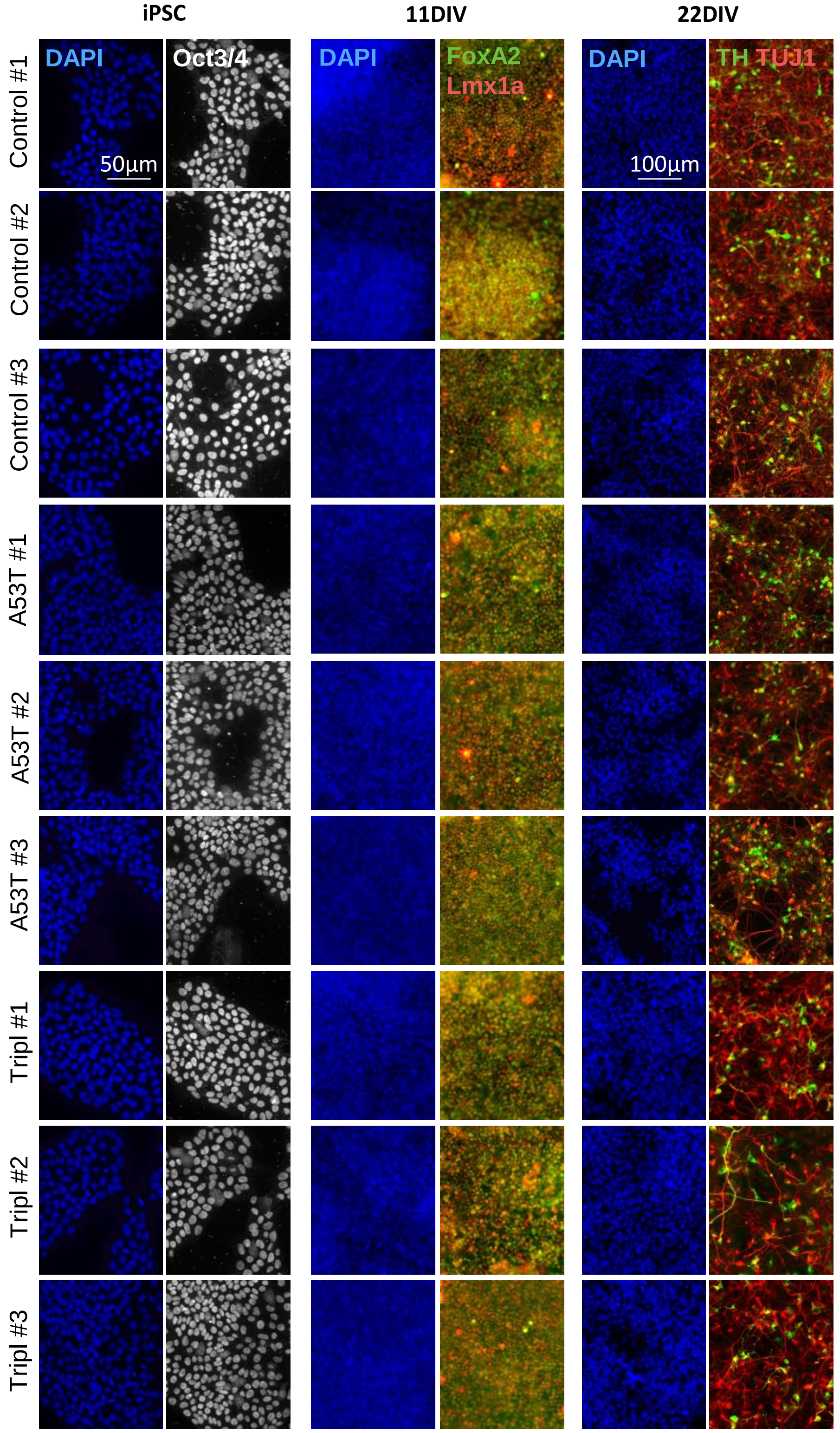

Supplementary Figure 1

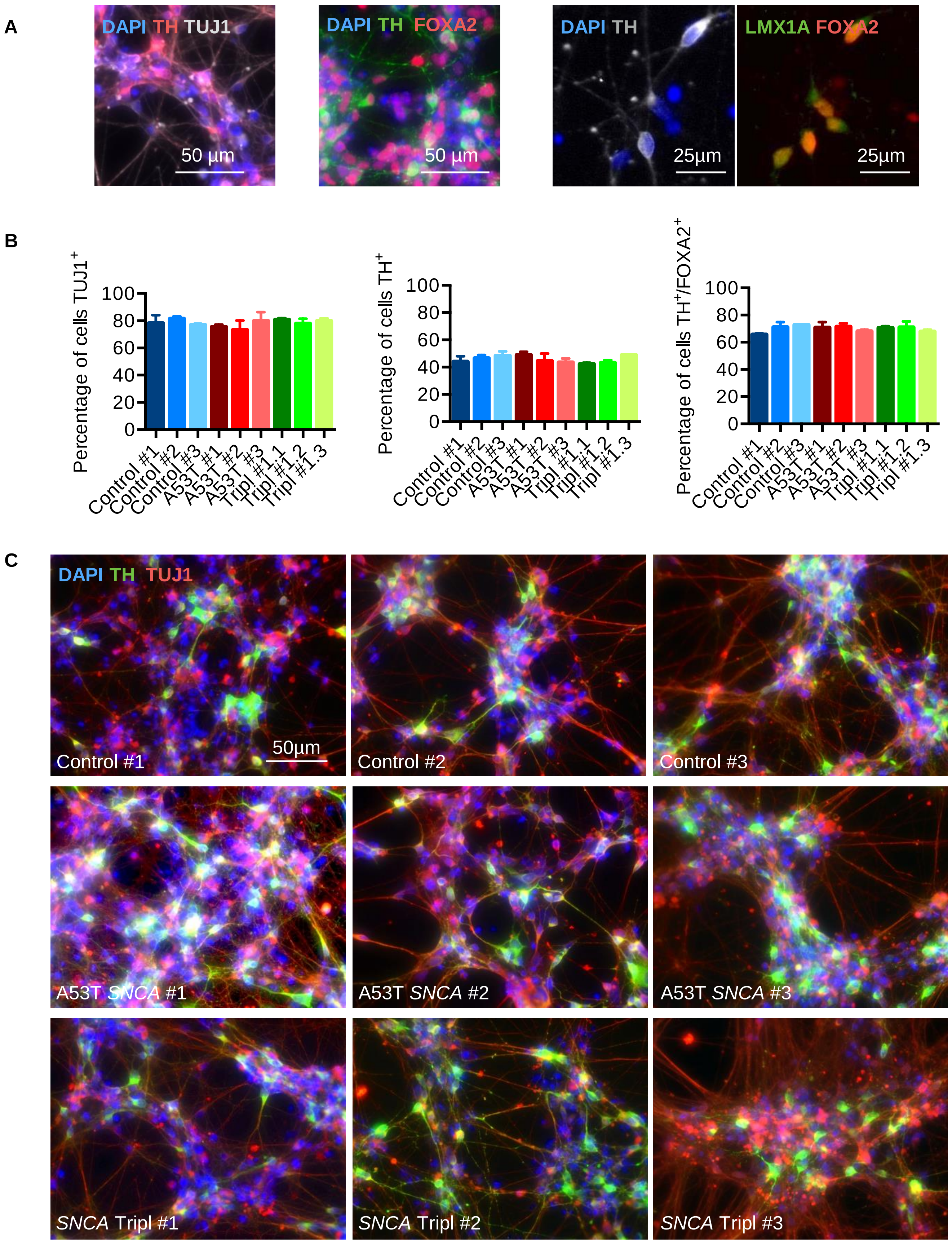

Supplementary Figure 2

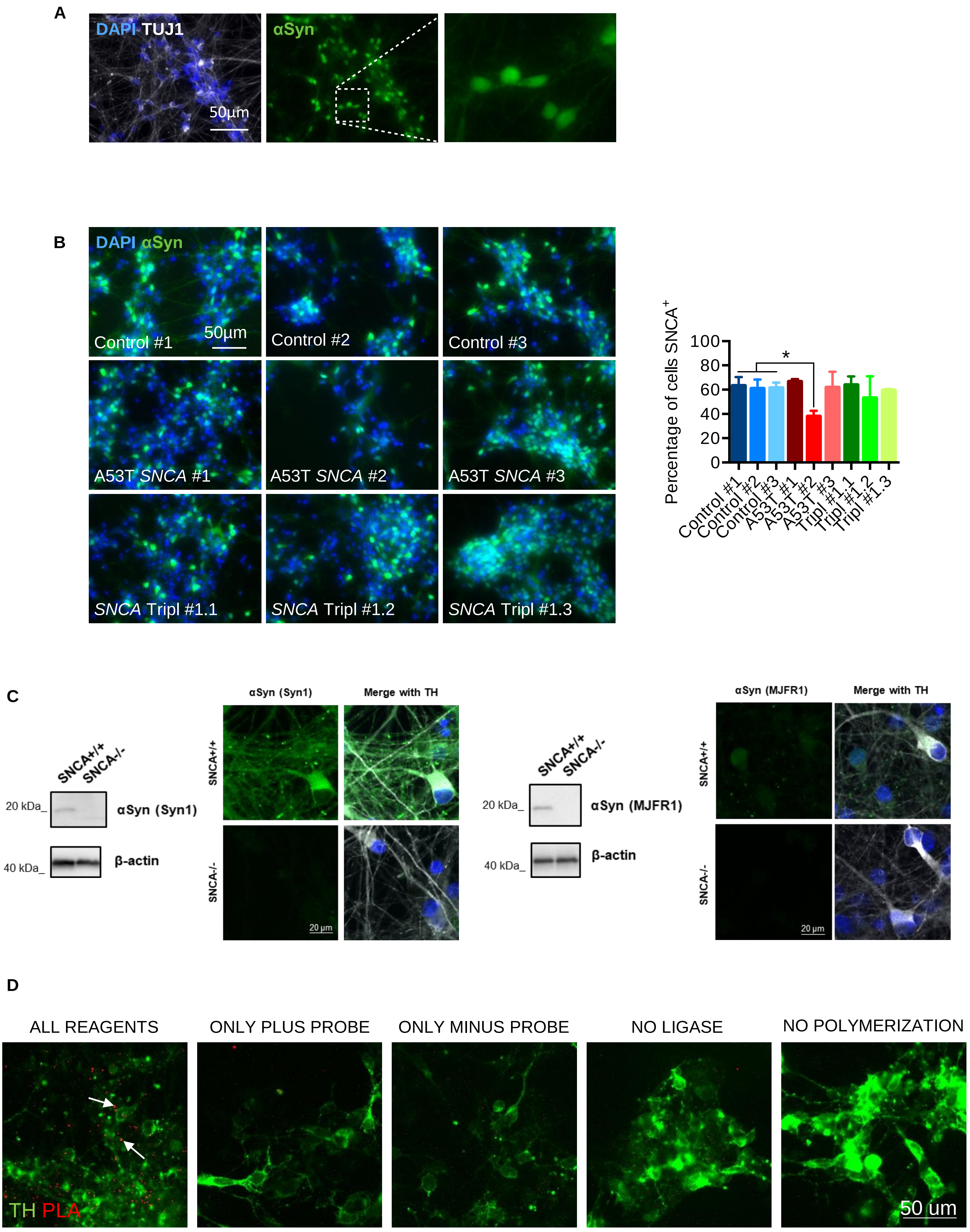

Supplementary Figure 3

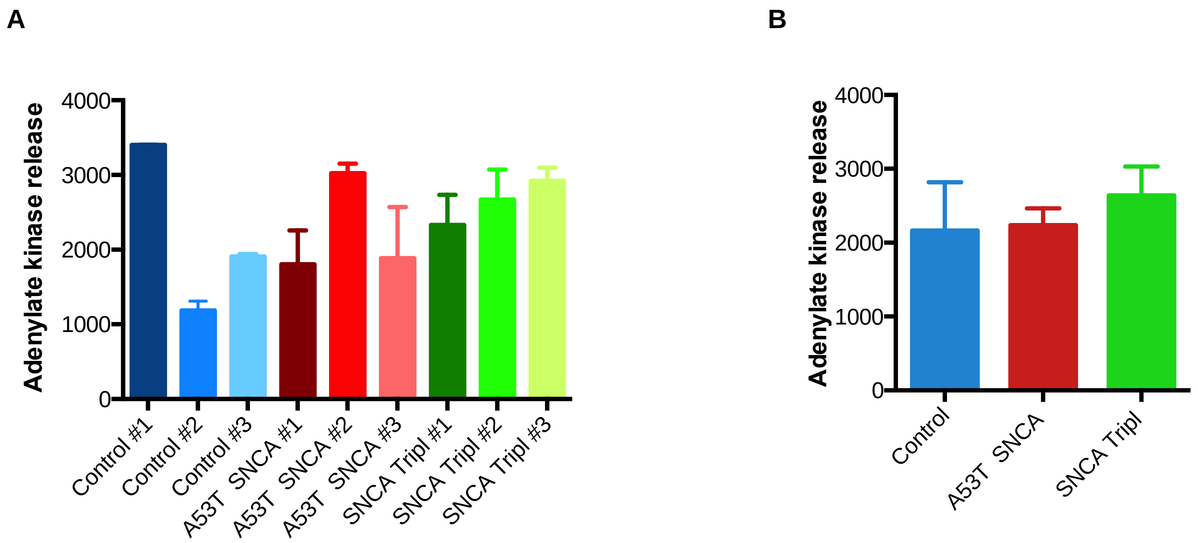

Supplementary Figure 4

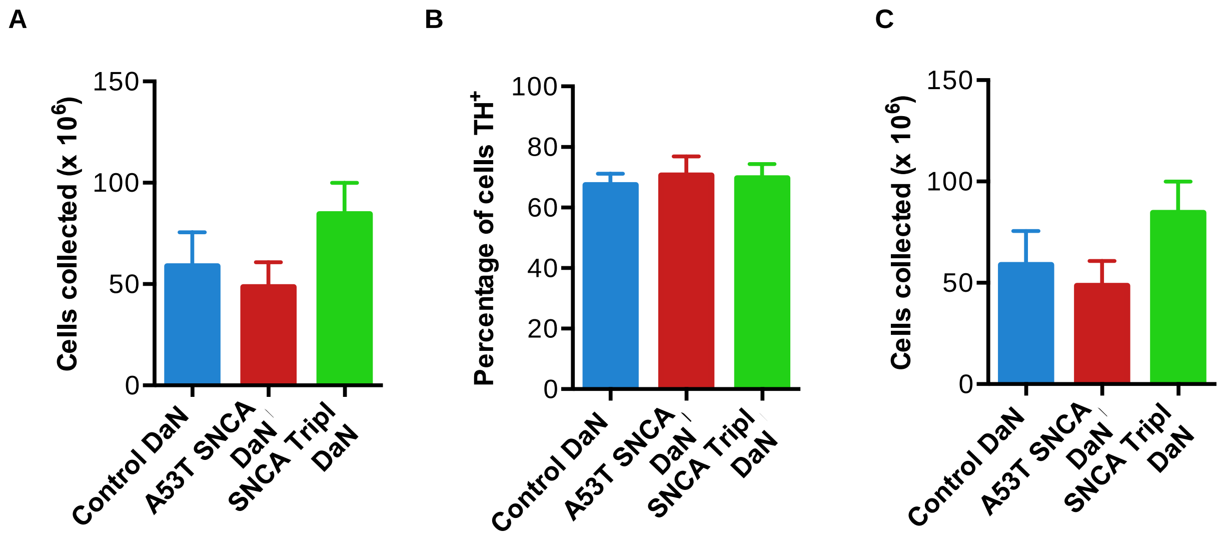

Supplementary Figure 5

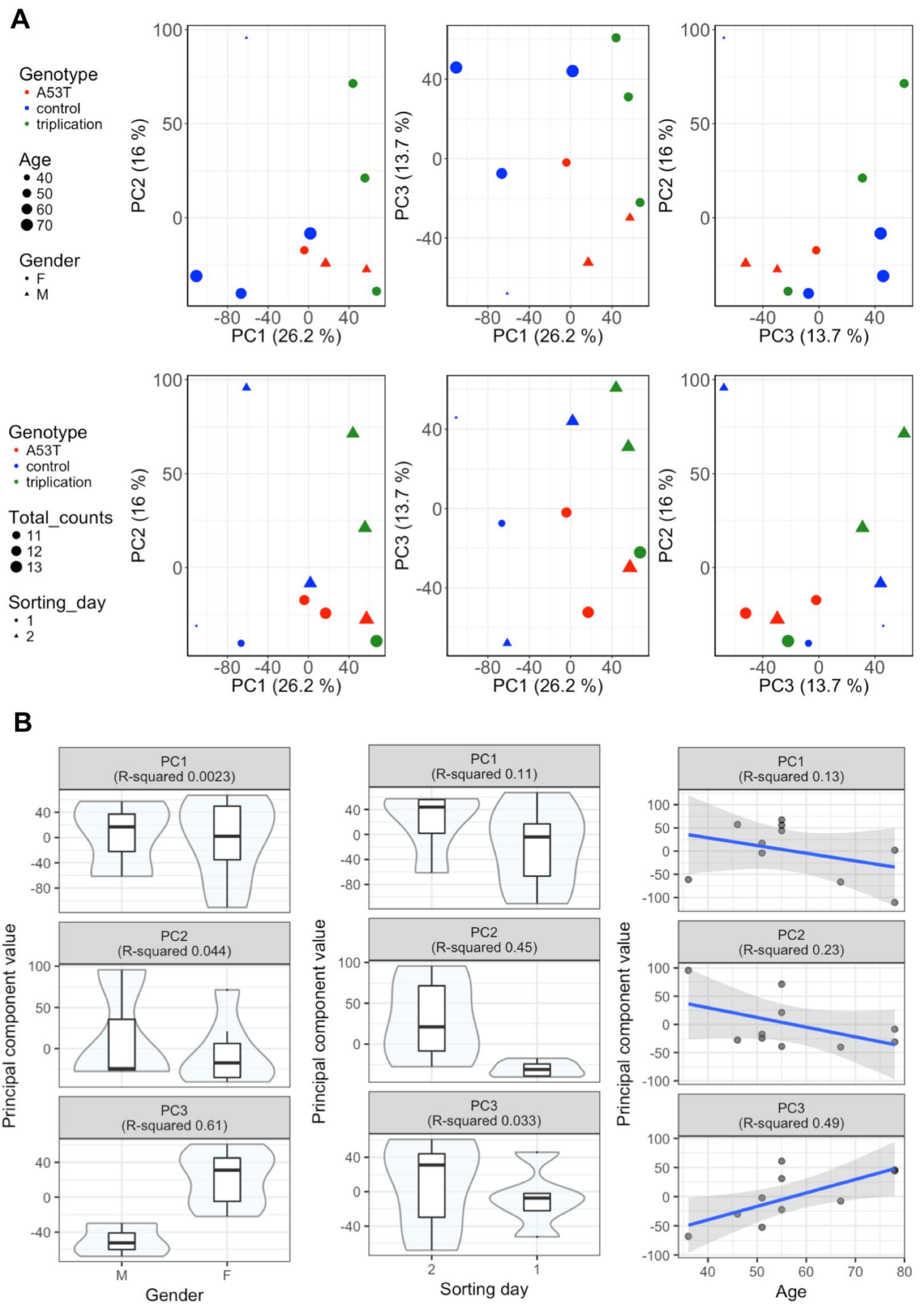

Supplementary Figure 6

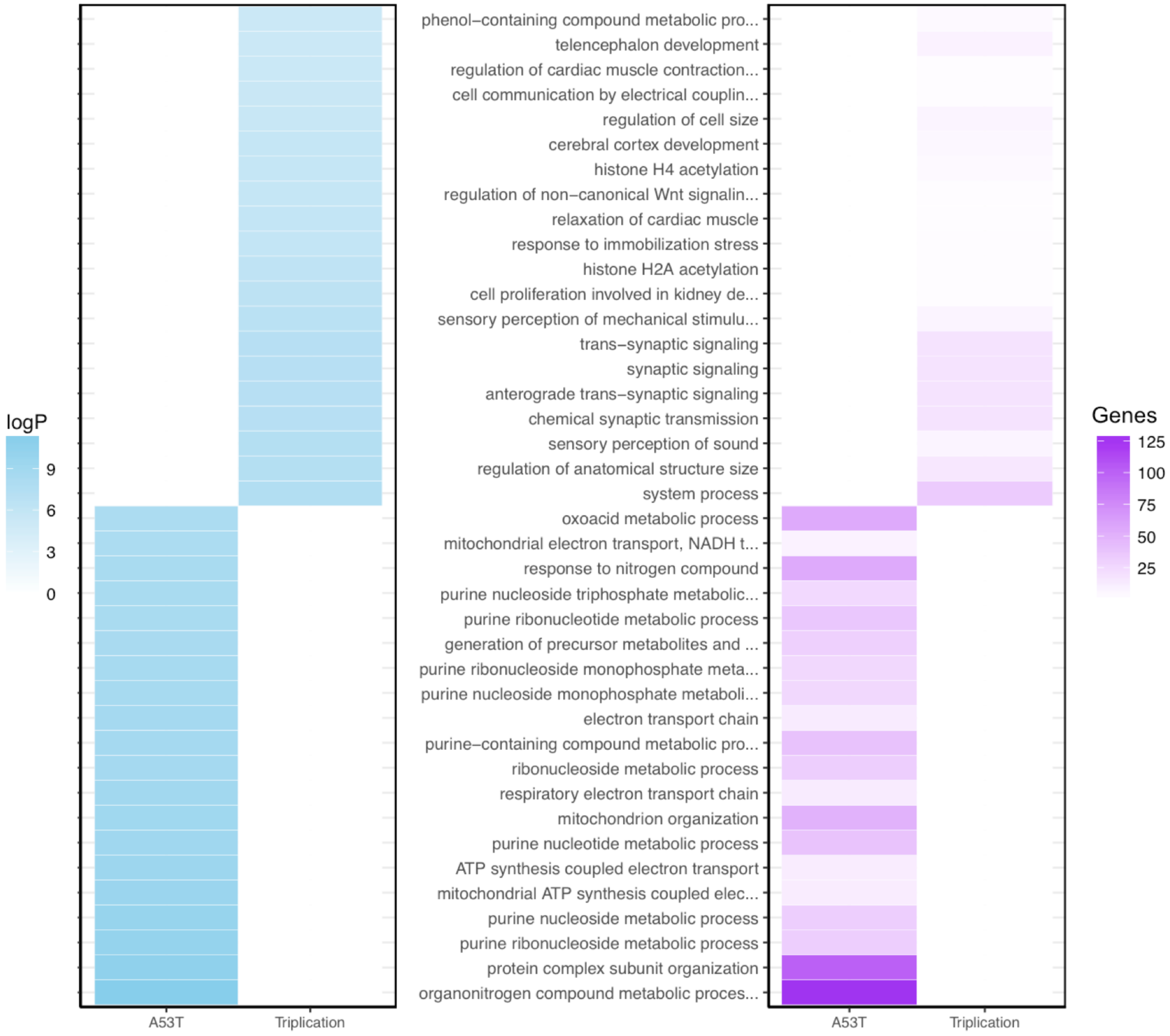

Supplementary Figure 7

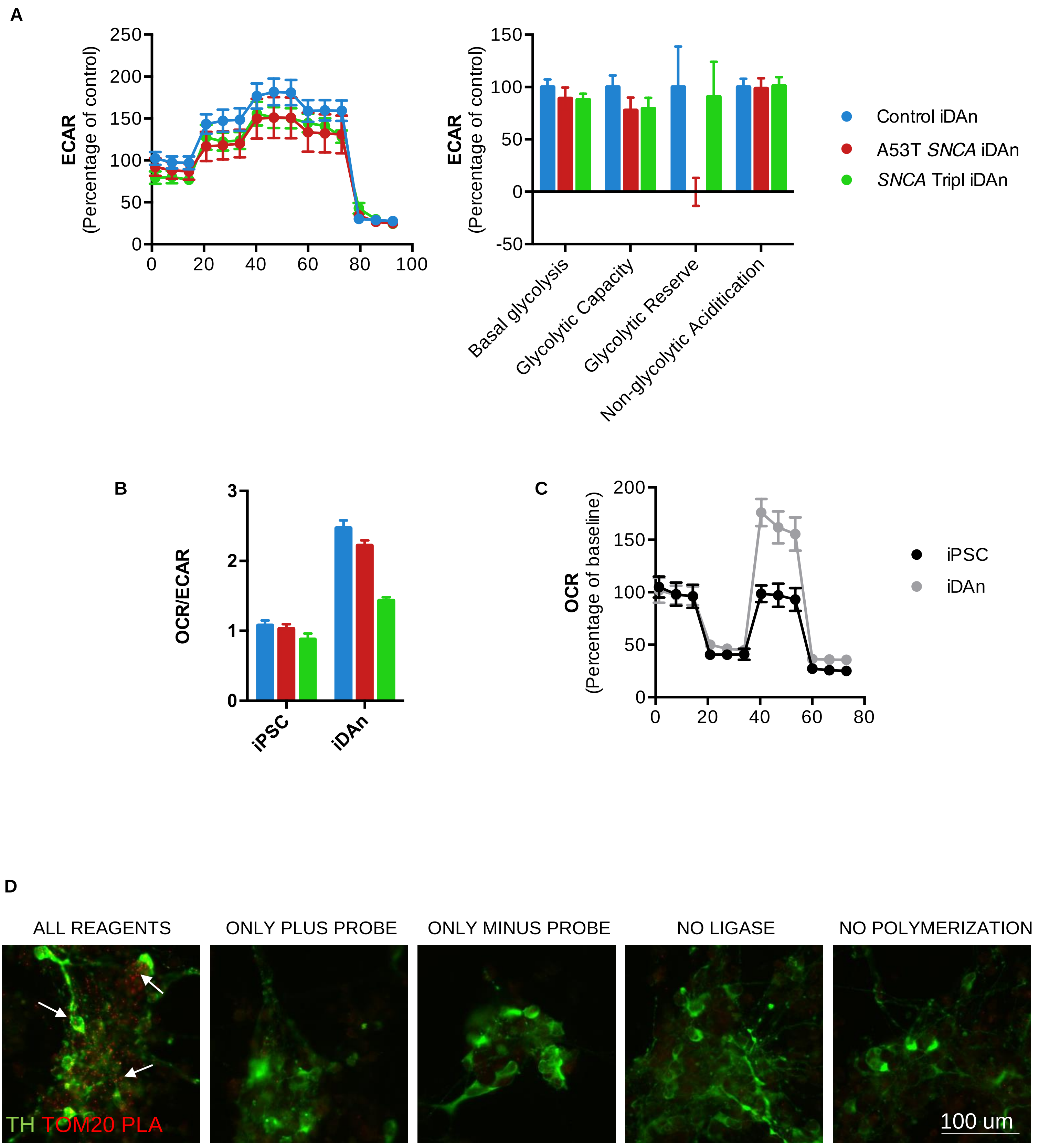

Supplementary Figure 8

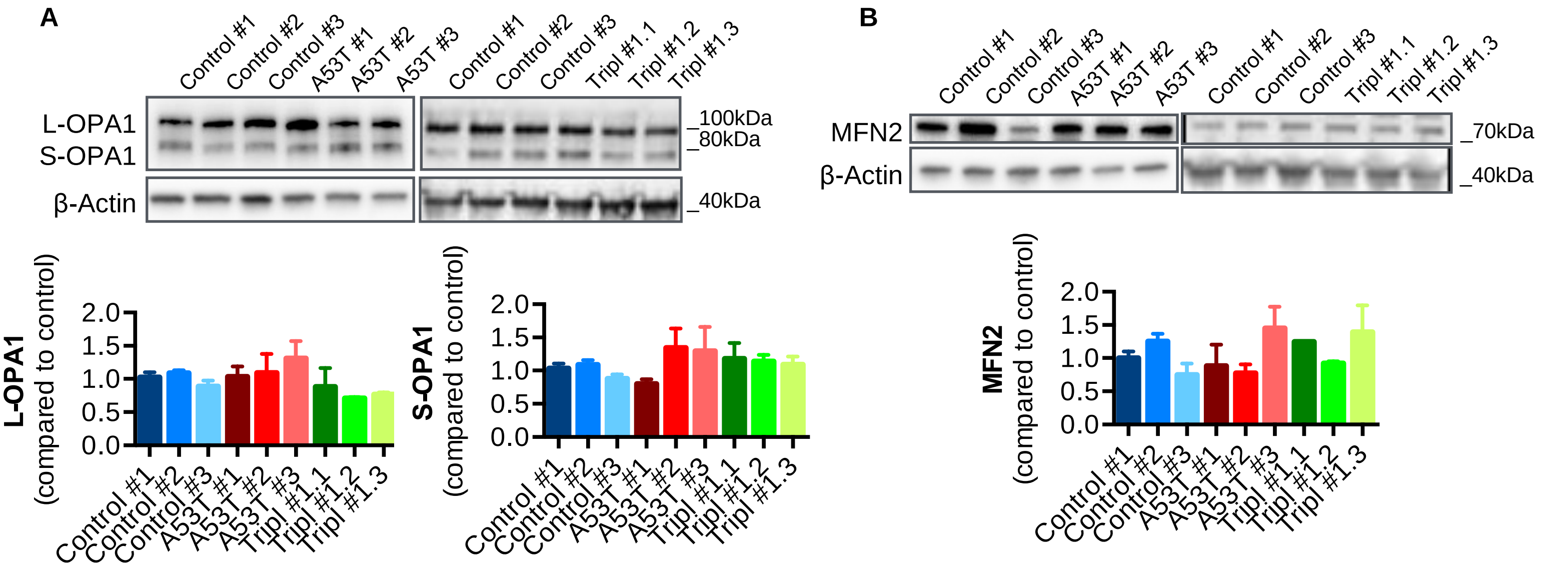

Supplementary Figure 9

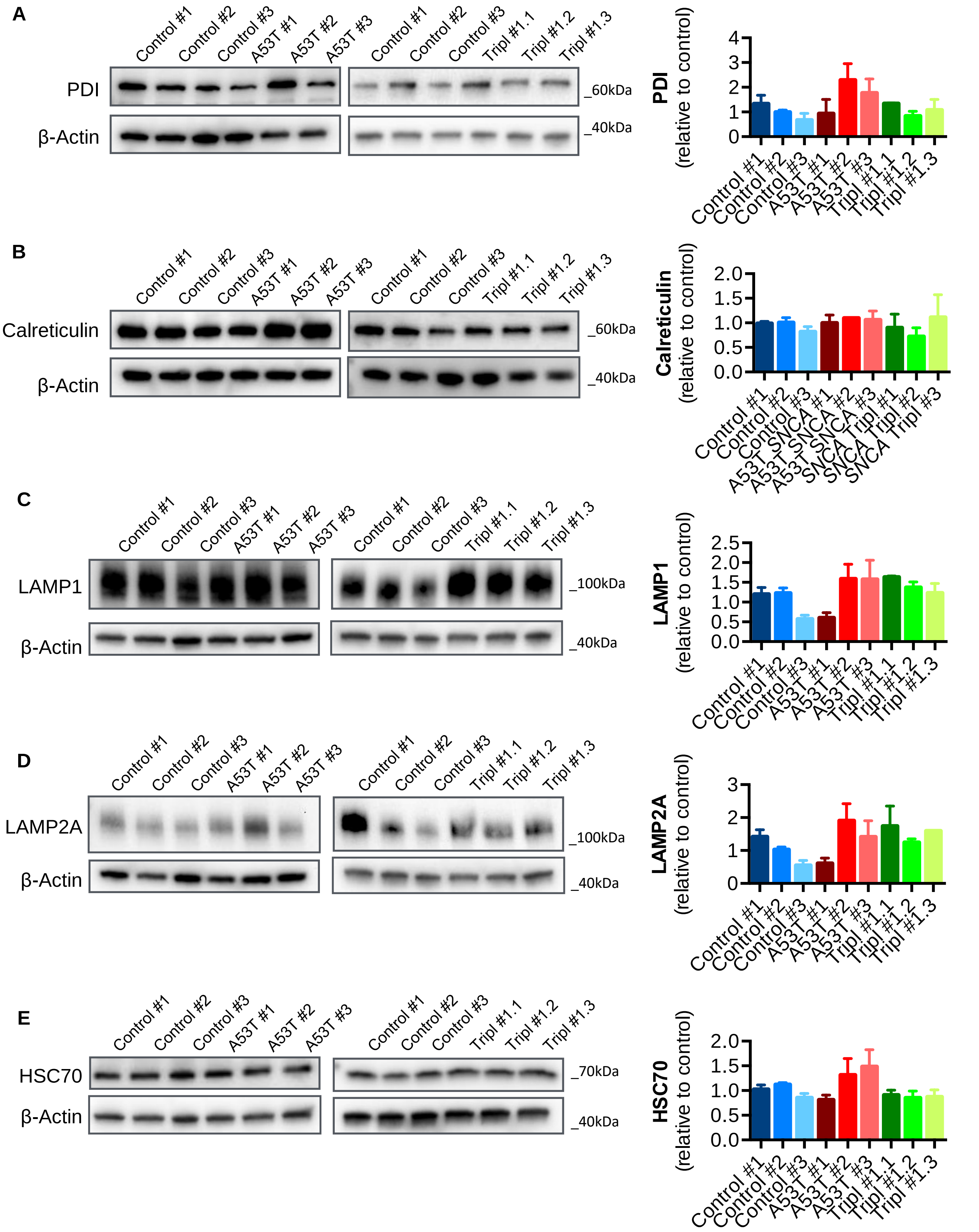

Supplementary Figure 10

A

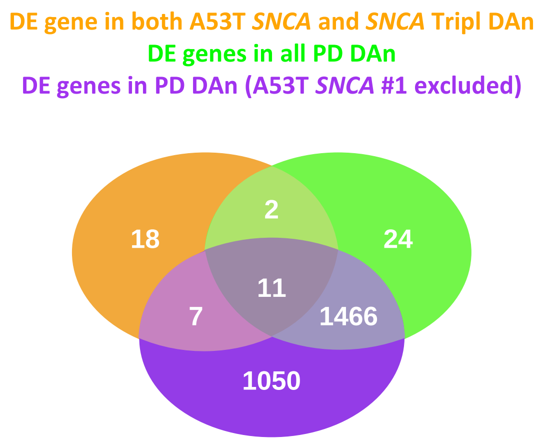

B

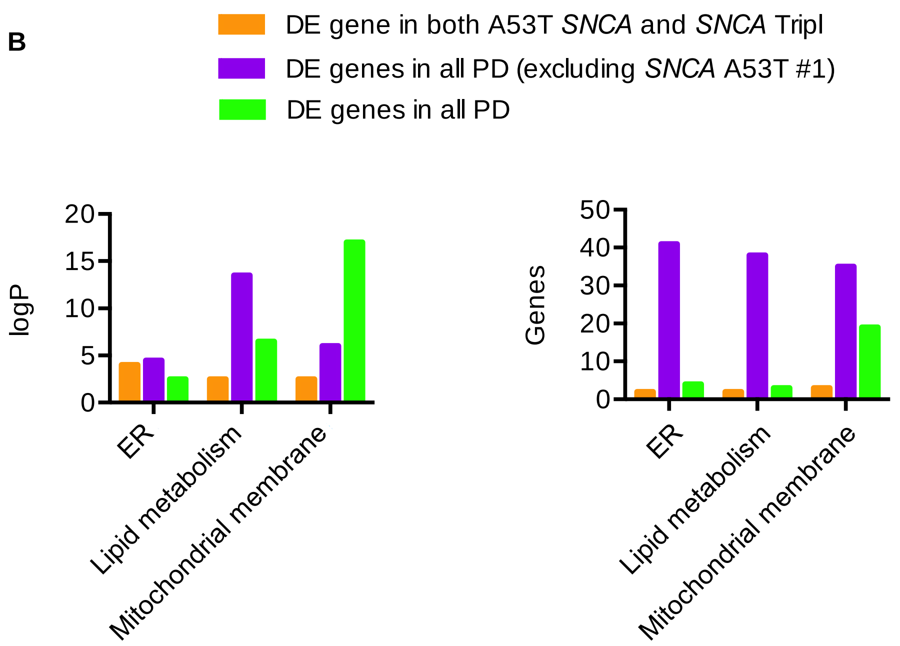

Supplementary Figure 11

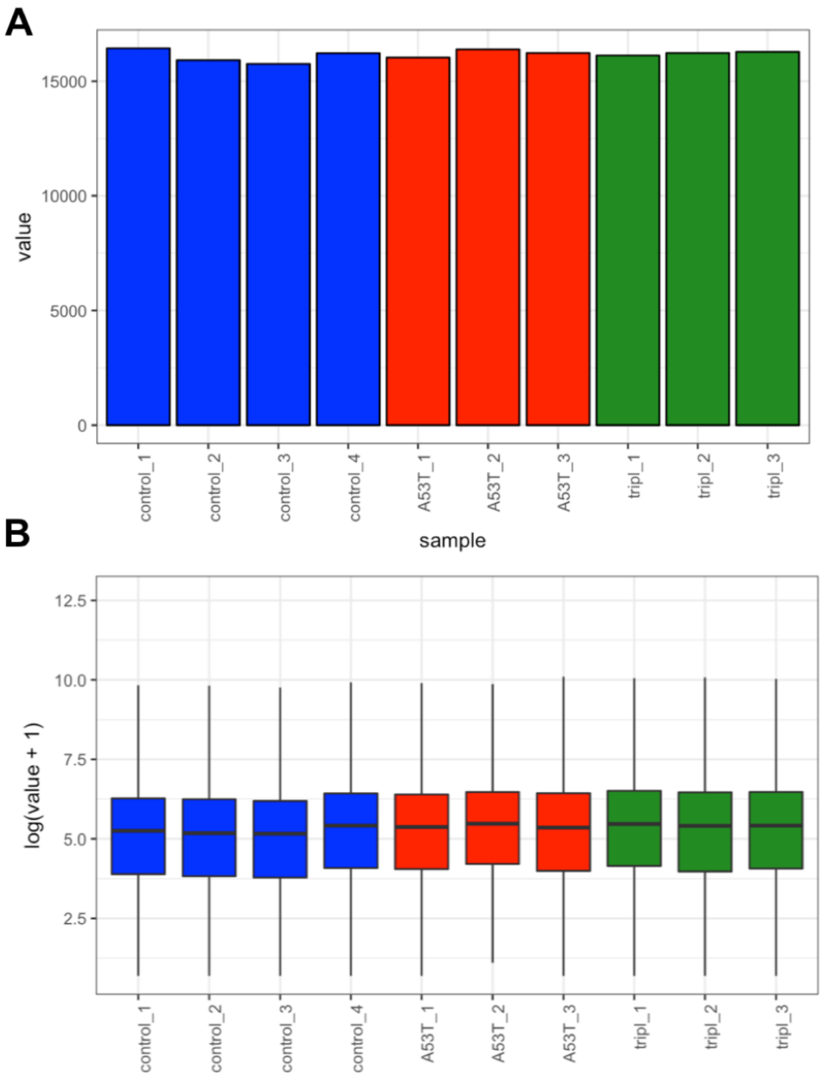

Supplementary Figure 12

	GO.ID	Term	Annotated	Significant	Expected	classic
1	GO:0097458	neuron part	1088	10	2.97	0.00048
2	GO:0031941	filamentous actin	21	2	0.06	0.00148
3	GO:0044421	extracellular region part	2630	14	7.19	0.00666
4	GO:0043230	extracellular organelle	2086	12	5.7	0.00719
5	GO:1903561	extracellular vesicle	2086	12	5.7	0.00719
6	GO:0005576	extracellular region	2957	15	8.08	0.00742
7	GO:0031988	membrane-bounded vesicle	2687	14	7.35	0.00811
8	GO:0031982	vesicle	2790	14	7.63	0.01137
9	GO:0005884	actin filament	68	2	0.19	0.01477
10	GO:0005746	mitochondrial respiratory chain	71	2	0.19	0.01603
11	GO:0042995	cell projection	1501	9	4.1	0.01701
12	GO:0070469	respiratory chain	77	2	0.21	0.01869
13	GO:0070062	extracellular exosome	2076	11	5.68	0.01902
14	GO:0043005	neuron projection	813	6	2.22	0.02149
15	GO:0000813	ESCRT I complex	10	1	0.03	0.02701
16	GO:0036513	Derlin-1 retrotranslocation complex	11	1	0.03	0.02967
17	GO:0000153	cytoplasmic ubiquitin ligase complex	13	1	0.04	0.03498
18	GO:0098590	plasma membrane region	686	5	1.88	0.03729
19	GO:0016020	membrane	6381	23	17.44	0.04279
20	GO:0044456	synapse part	500	4	1.37	0.04628
21	GO:0098794	postsynapse	297	3	0.81	0.04686
22	GO:0030426	growth cone	129	2	0.35	0.04829
23	GO:0031252	cell leading edge	305	3	0.83	0.05003
24	GO:0070069	cytochrome complex	19	1	0.05	0.05072
25	GO:0030427	site of polarized growth	133	2	0.36	0.05099
26	GO:0044425	membrane part	4351	17	11.89	0.0524
27	GO:0001726	ruffle	136	2	0.37	0.05306
28	GO:0098858	actin-based cell projection	138	2	0.38	0.05446
29	GO:0009925	basal plasma membrane	22	1	0.06	0.05849
30	GO:1902555	endoribonuclease complex	22	1	0.06	0.05849
31	GO:0036477	somatodendritic compartment	547	4	1.5	0.06067
32	GO:0030027	lamellipodium	148	2	0.4	0.06162
33	GO:0044463	cell projection part	797	5	2.18	0.0637
34	GO:0032281	AMPA glutamate receptor complex	25	1	0.07	0.06621
35	GO:0036452	ESCRT complex	27	1	0.07	0.07132
36	GO:0098796	membrane protein complex	827	5	2.26	0.07231
37	GO:0032420	stereocilium	30	1	0.08	0.07893
38	GO:0044455	mitochondrial membrane part	171	2	0.47	0.07923
39	GO:0044459	plasma membrane part	1686	8	4.61	0.08068
40	GO:0031093	platelet alpha granule lumen	31	1	0.08	0.08145
41	GO:0015629	actin cytoskeleton	375	3	1.03	0.08196
42	GO:0045211	postsynaptic membrane	177	2	0.48	0.08405
43	GO:0032421	stereocilium bundle	33	1	0.09	0.08648
44	GO:0034707	chloride channel complex	33	1	0.09	0.08648
45	GO:0045178	basal part of cell	34	1	0.09	0.08898
46	GO:0045202	synapse	632	4	1.73	0.09212
47	GO:0030425	dendrite	395	3	1.08	0.0924
48	GO:0045121	membrane raft	201	2	0.55	0.10421
49	GO:0098857	membrane microdomain	202	2	0.55	0.10508

Supplementary Table 1

	GO.ID	Term	Annotated	Significant	Expected	classic
50	GO:0016328	lateral plasma membrane	41	1	0.11	0.10632
51	GO:0008328	ionotropic glutamate receptor complex	42	1	0.11	0.10878
52	GO:0098878	neurotransmitter receptor complex	42	1	0.11	0.10878
53	GO:0034702	ion channel complex	209	2	0.57	0.11121
54	GO:0005747	mitochondrial respiratory chain complex ...	43	1	0.12	0.11122
55	GO:0016459	myosin complex	43	1	0.12	0.11122
56	GO:0030964	NADH dehydrogenase complex	43	1	0.12	0.11122
57	GO:0031519	PcG protein complex	43	1	0.12	0.11122
58	GO:0045271	respiratory chain complex I	43	1	0.12	0.11122
59	GO:0005743	mitochondrial inner membrane	433	3	1.18	0.11369
60	GO:0031091	platelet alpha granule	46	1	0.13	0.11851
61	GO:0005882	intermediate filament	48	1	0.13	0.12334
62	GO:0030141	secretory granule	225	2	0.62	0.12556
63	GO:0005902	microvillus	49	1	0.13	0.12575
64	GO:0034774	secretory granule lumen	49	1	0.13	0.12575
65	GO:0043235	receptor complex	227	2	0.62	0.12738
66	GO:0045177	apical part of cell	229	2	0.63	0.12921
67	GO:0097060	synaptic membrane	229	2	0.63	0.12921
68	GO:0044433	cytoplasmic vesicle part	462	3	1.26	0.13109
69	GO:0005886	plasma membrane	3160	12	8.64	0.13185
70	GO:0001750	photoreceptor outer segment	52	1	0.14	0.13293
71	GO:0005811	lipid particle	52	1	0.14	0.13293
72	GO:0005901	caveola	52	1	0.14	0.13293
73	GO:0099080	supramolecular complex	466	3	1.27	0.13357
74	GO:0099081	supramolecular polymer	466	3	1.27	0.13357
75	GO:0099512	supramolecular fiber	466	3	1.27	0.13357
76	GO:0099513	polymeric cytoskeletal fiber	466	3	1.27	0.13357
77	GO:0030672	synaptic vesicle membrane	53	1	0.14	0.13531
78	GO:0099501	exocytic vesicle membrane	53	1	0.14	0.13531
79	GO:1902495	transmembrane transporter complex	237	2	0.65	0.1366
80	GO:0019866	organelle inner membrane	477	3	1.3	0.14046
81	GO:1990351	transporter complex	243	2	0.66	0.14221
82	GO:0043195	terminal bouton	56	1	0.15	0.14241
83	GO:0060205	cytoplasmic membrane-bounded vesicle lum...	58	1	0.16	0.14711
84	GO:0031983	vesicle lumen	59	1	0.16	0.14946
85	GO:0044853	plasma membrane raft	59	1	0.16	0.14946
86	GO:0030667	secretory granule membrane	60	1	0.16	0.15179
87	GO:0071944	cell periphery	3242	12	8.86	0.15231
88	GO:0098803	respiratory chain complex	63	1	0.17	0.15876
89	GO:0043234	protein complex	3270	12	8.94	0.1597
90	GO:0005903	brush border	65	1	0.18	0.16338
91	GO:0032587	ruffle membrane	67	1	0.18	0.16797
92	GO:0098589	membrane region	270	2	0.74	0.16801
93	GO:0048471	perinuclear region of cytoplasm	533	3	1.46	0.17733
94	GO:0098805	whole membrane	1112	5	3.04	0.18352
95	GO:0005643	nuclear pore	74	1	0.2	0.18385
96	GO:0005604	basement membrane	76	1	0.21	0.18833
97	GO:0031225	anchored component of membrane	78	1	0.21	0.19279
98	GO:0044444	cytoplasmic part	6566	21	17.95	0.19417
99	GO:0030175	filopodium	79	1	0.22	0.19501
100	GO:0031224	intrinsic component of membrane	3433	12	9.38	0.20657

Supplementary Table 1
