# Supplementary material for: Cellular α-synuclein pathology is associated with bioenergetic dysfunction in Parkinson’s iPSC-derived dopamine neurons
Source: Hum Mol Genet. 2019 Feb 11;28(12):2001–13. doi: 10.1093/hmg/ddz038 (PMC6548224; doi:10.1093/hmg/ddz038)
Supplement: Supplementary Data [file suppl_data_ddz038.zip › Zambon et al. - Supplementary_Dec.docx]

**SUPPLEMENTARY FIGURE LEGENDS**

**Supplementary Figure 1: Characterisation of control, A53T *SNCA* and *SNCA* Tripl iPSC differentiation**

(A) List of all iPSC lines used in this study. (B) iPSC lines stain positive for the pluripotency marker Oct 3/4. On 11DIV, most cells express the FOXA2 and LMX1A markers, indicating successful midbrain patterning. On 22DIV, a large proportion of cells expresses TUJ1 and TH, indicating successful generation of DAn.

**Supplementary Figure 2: Characterisation of iPSC-derived DAn at 35DIV.**

(A) Representative images of TUJ1, TH and FOXA2 immuno-staining. (B) Quantification of the percentage of neurons (TUJ1^+^), dopaminergic neurons (TH^+^) and FOXA2^+^ dopaminergic neurons (TH^+^/FOXA2^+^) (N=3, mean ± SEM). (C) Representative images of control, A53T *SNCA* and *SNCA* Tripl DAn were fixed at 35DIV and stained for TUJ1 and TH.

**Supplementary Figure 3: αSyn immunocytochemistry in iPSC-derived DAn.**

(A) αSyn immunocytochemistry in DAn cultures. (B) Representative images of αSyn immuno-staining and quantification of the percentage of cells expressing αSyn (N=3, mean ± SEM, one-way ANOVA, **p*<0.05). (C) Representative images of Syn1 and MJFR1 αSyn immuno-staining and immunoblotting to confirm lack of expression in *SNCA* knockout (KO) iPSC-derived DAn (DIV40). (D) Complete and incomplete control PLA reactions for aSyn 4D6 was achieved by introducing and removing specific components (plus/minus probe, ligase and polymerization mix) of the assay. White arrows indicate PLA puncta.

**Supplementary Figure 4: Adenylate kinase release from iPSC-derived DAn.**

Adenylate kinase release from DAn cultures. Adenylate kinase was measured in the medium from 35DIV DAn to assess stress-induced protein secretion from cultures. Adenylate kinase release from (A) individual patients (n=3; ANOVA P>0.05 +/- SEM) and (B) by genotype demonstrating no significant difference in adenylate kinase release between genotypes (N=3; ANOVA P>0.05 +/- SEM).

**Supplementary Figure 5: Quality control of FACS and RNA extraction from iPSC-derived DAn.**

(A) Total number of TH^+^ cells collected during FACS of TH-labelled FACS of DAn cultures. (B) Percentage of TH^+^ cells compared to total cells during FACS of TH-labelled FACS of DAn cultures. (C) RIN of RNA extracted from FACS of TH^+^ cells from DAn cultures.

**Supplementary Figure 6: PCA plots and correlations between principal components and known covariates**

(A) PCA plots on 14.214 coding genes expressed in at least half of the samples. All known covariates are shown with different size and shape while colours are indicative of genotypes. B) The first three principal components are plotted against known covariates (gender, sorting day and age) and the R^2^ value from a linear model regressing each PC onto the variable of interest is reported. PC3 correlates with gender and age, PC2 correlates with sorting day.

**Supplementary Figure 7: Gene Ontology enrichment for genes DE between A53T *SNCA* and *SNCA* Tripl iPSC-derived DAn compared to control**

The top 20 enriched Gene Ontology Biological Process terms in DE genes in A53T *SNCA* and in *SNCA* Tripl compared to control. The statistical significance of enrichment (absolute log10 p-value) is shown on the left and the number of significant DE genes found in each term is shown on the right.

**Supplementary Figure 8: Glycolytic activity and OCR/ECAR ratio in iPSC and iPSC-derived DAn cultures**

(A) ECAR traces and glycolytic activity parameters in DAn (N=3, mean ± SEM); the average value for the glycolytic reserve is -0.057±13.1386. (B) OCR/ECAR ratio in iPSCs and DAn. (C) Comparison of the OCR trace between iPSCs and DAn. (D) Complete and incomplete PLA for aSyn 4D6-TOM20 was achieved by introducing and removing specific components (plus/minus probe, ligase and polymerization mix) of the assay. White arrows indicate PLA puncta.

**Supplementary Figure 9: Changes in mitochondrial fission/fusion proteins in *SNCA* A53T and *SNCA* Tripl iPSC-derived DAn.**

(A) L-OPA1 and S-OPA1 protein levels in DAn (N=3, mean ± SEM, one-way ANOVA, **p*<0.05, ***p*<0.01). (B) MFN2 protein levels in DAn. In each case, data represent the mean ± SEM from three independent differentiation (N=3). One-way ANOVA, **p*<0.05, ***p*<0.01.

**Supplementary Figure 10: Lysosomal, CMA and other ER markers are unchanged in *SNCA* A53T and *SNCA* Tripl iPSC-derived DAn.**

(A) PDI protein levels in DAn. (B) Calreticulin protein levels in DAn. (C) LAMP1 protein levels in DAn. (D) LAMP2A protein levels in DAn. (E) HSC70 protein levels in DAn. In each case, data represent the mean ± SEM from three independent differentiation (N=3).

**Supplementary Figure 11: Removal of A53T *SNCA* #1 from the transcriptomics analysis**

(A) Venn Diagram of 38 common DE genes in A53T and triplication compared to controls, 1503 DE genes in all six PD lines compared to controls and 2534 DE genes in five PD lines (excluding A53T *SNCA* #1) compared to controls. (B) Pathway analysis for different sets of DE genes focussed on phenotypes characterised in the previous sections (ER, lipid metabolism and mitochondria -related terms). The significance of enrichment is reported on y axis (absolute log10 p-value) and colours represent different sets of DE genes. The number of genes found in each term is reported on y axis and colours represent different sets of DE genes.

**Supplementary Figure 12: Preliminary analysis of RNA-seq data**

(A) Number of expressed genes (count>=1). The largest difference is of about 700 genes. (B) Boxplots of average expression values for all genes. No outliers are detected.

**Supplementary Table 1: Complete list of enriched GO terms**

**SUPPLEMENTARY EXPERIMENTAL PROCEDURES**

**Differentiation of iPSCs to DAn**iPSCs cells were patterned for 11 days to become ventral midbrain precursors cells with LDN193189 (100nM, Sigma-Aldrich), SB431542 (10 mM, Tocris), SHH C24II N-terminus (100 ng ml^-1^, Bio-Techne), Purmorphamine (2 mM, Millipore), FGF8a (100 ng ml^-1^, Bio-Techne) and CHIR99021 (3 mM, Tocris). Two days prior neuronal induction, iPSCs were dissociated to single cells and plated at a density of 150,000 cells/cm^2^ on Geltrex (Life Technologies) and cultured until sub-confluent. Cells were then grown 11 days in knockout serum replacement medium (KSR) containing KO DMEM (Life Technologies), 15% knockout serum replacement (Life Technologies), 2 mM L-glutamine (Life Technologies) and 10 mM β-mercaptoethanol (Sigma-Aldrich). KSR medium was gradually shifted to NNB medium containing Neurobasal medium, 0.5X N2, 0.5X B27 and 2 mM L-glutamine (Life Technologies) starting on day 5 of differentiation as described previously. On day 11, media was changed to NB medium containing Neurobasal medium, B27 and 2 mM L-glutamine (Life Technologies) supplemented with CHIR (until day 13) and with BDNF (brain-derived neurotrophic factor, 20n ml^-1^; Peprotech), ascorbic acid (0.2 mM, Sigma-Aldrich), GDNF (glial cell line-derived neurotrophic factor, 20 ng ml^-1^; Peprotech), TGFβ3 (transforming growth factor type β3, 1 ng ml^-1^; Peprotech), dibutyryl cAMP (0.5mM; Sigma-Aldrich), and DAPT (10mM; Abcam) for 9 days. On day 20, cells were dissociated using Accutase (Life Technologies) and replated at the cell density of 300,000 cells/cm^2^ in dishes pre-coated with Geltrex in final differentiation medium (NB supplemented with BDNF, GDNF, TGFb3, DAPT, dbcAMP and ascorbic acid). On day 22, cells were treated for an hour with 1 μg ml^-1^ of Mitomycin C (Life Technologies) in NB medium. Cells were then cultured in final differentiation medium for two weeks until day 35 before any given experiment.

**Immunocytochemistry**
Cells were fixed in 4% paraformaldehyde (PFA). Cells were permeabilised and blocked in PBS containing 0.1% Triton-X100 and 10% serum for 1 hour at RT, before incubation with primary antibodies overnight at 4°C in PBS containing 0.1% Triton-X100 and 1% serum. Antibodies used as follows: αSyn (Syn1 BD, #610786, 1:500, 4D6 Abcam ab1903, 1:250 or MJFR1 Abcam ab138501, 1:250), TH (Millipore, #AB152, 1:500), TUJ1 (Abcam, #ab107216, 1:1000), FOXA2 (R&D, #AF2400, 1:250), LMX1A (Millipore, #AB10533, 1:500). AlexaFluor-conjugated secondary antibodies were incubated for 1 hour at RT in PBS containing 0.1% Triton-X100 and 1% serum. Nuclear DNA was stained with DAPI (Thermo Fisher Scientific) for 5 minutes at RT. Coverslips were mounted onto microscope slides with FluorSave (Merk Millipore). Images were captured on a EVOS FL Auto Cell Imaging System (Life Technologies).

**Protein extraction and Western Blot analysis**Soluble proteins were extracted from cell pellets using RIPA buffer (50 mM Tris (pH 7.4), 150 mM NaCl, 1% v/v Triton X-100, 1% w/v sodium deoxycholate and 0.1% w/v SDS) supplemented with cOmplete protease inhibitor cocktail (Sigma-Aldrich-Aldrich) and PhosSTOP (Sigma-Aldrich-Aldrich). Protein content was quantified by Bicinchoninic acid (BCA) assay. Samples for gel electrophoresis were prepared by adding 5X Laemmli Buffer (0.1% 2-Mercaptoethanol, 0.0005% w/v bromophenol blue, 10% v/v glycerol, 2% w/v SDS and 63mM Tris-HCl pH 6.8), before being denatured at 95°C for 10 minutes. 5-10 μg of protein were separated using Tris-Glycine gels (Bio-Rad) before being transferred onto PVDF membranes (Bio-Rad) using a Trans-Blot^®^ TurboTM Transfer System (Bio-Rad). Membranes were blocked with 5% skimmed milk in 0.1% Tween 20/TBS for an hour. Primary antibodies were incubated overnight at 4°C at the appropriate dilution in 1% skimmed milk in Tween 20/TBS. Antibodies used as follows: αSyn (Syn1 BD, #610786, 1:1000, 4D6 Abcam ab1903, 1:500 or MJFR1 Abcam ab138501, 1:1000), Catalase (Cell Signalling, #12980, 1:1000), CYP46A1 (ProteinTech, #12486-1-AP, 1:1000), FABP7 (Cell Signalling, #13347, 1:1000), SIRT1 (Cell Signalling, #2496, 1:1000), BiP (Abcam, #ab21685, 1:1000), Calreticulin (Abcam, #ab108395, 1:1000), IRE1α (Cell Signalling, #3294, 1:1000), PDI (Cell Signalling, #3501, 1:1000), LAMP2A (Abcam, #ab18528, 1:1000), LAMP1 (Santa Cruz, #sc-20011, 1:200), LC3B (Sigma, #L7534, 1:1000), p62 (Abcam, #ab109012, 1:1000), DRP1 (Cell Signalling, #8570, 1:1000), phospho-DRP1^Ser616^ (Cell Signalling, #4494, 1:1000), MFN2 (Sigma, #M6319, 1:1000), OPA1 (BD, #612606, 1:1000), PGC-1α (Santa Cruz, #sc-13067, 1:100), PHB (Abcam, #ab28172, 1:1000), TOM20 (Santa Cruz, #sc-11415, 1:1000). HRP-conjugated secondary antibodies (Bio-Rad) were used at the dilution of 1:5000 and incubated with the membrane for 1 hour. The Immobilon Western Chemiluminescent HRP Substrate (Millipore) was used to develop the membrane and images were acquired using a Gel DocTM XR+ System (Bio-Rad). Densitometry analysis was performed with ImageJ (NIH, Bethesda, Maryland, USA) to quantify bands; these were then normalised for the loading control β-actin (Abcam, #ab49900, 1:20000).

**Measurement of mitochondria respiration using the Seahorse Analyzer**
Cells were plated in a XF96 Polystyrene Cell Culture Microplate (Seahorse Bioscience) on day 20 and further matured until day 35 before analysis. Alternatively, iPSCs were plated the day before the experiment. On the day of the assay, the assay medium was prepared fresh using the XF Base Medium (Seahorse Bioscience) supplemented with 10 mM Glucose (Sigma-Aldrich-Aldrich), 1 mM Sodium Pyruvate (Sigma-Aldrich-Aldrich) and 2 mM L-Glutamine (Thermo Fisher Scientific). One hour before the assay, the cells were washed once with the assay medium and then incubated at 37°C in a non-CO_2_ incubator. Three baseline recordings were made, followed by sequential injection of the ATP synthase inhibitor oligomycin (Sigma-Aldrich), the mitochondrial uncoupler p-triflouromethoxyphenylhydrazone (FCCP; Sigma-Aldrich) and the Complex I and III inhibitors Rotenone and Antimycin A (Sigma-Aldrich). Final data were normalised to protein content in each well at the time of assay and analysed according to the manufacturer’s guidelines.

**FACS of TH^+^ cells from DAn cultures and RNA extraction for RNA-seq**
Briefly, DAn were dissociated in trypsin-EDTA and collected in PBS with DNAse I. Cells were stained using a live/dead yellow fixable stain (Life Technologies) before PFA fixation. Cells were then incubated with a primary TH antibody or a normal IgG2a antibody for 1 h on ice, prior quick permeabilisation with a saponin and RNAse inhibitor buffer. Cells were then incubated with a fluorescent secondary antibody on ice for 15 minutes. Samples were then sorted using FACS and TH^+^ and TH^-^ were collected and stored in 2ml Eppendorf DNA LoBind. RNA was extracted using an RNeasy micro kit (Qiagen) with minor alterations. RNA integrity (RIN) and concentration were analysed on a 2100 Bioanalyzer system (Agilent) utilizing the RNA 6000 pico kit (Agilent), following manufacturers’ instructions.
